# Does the host matter? Testing the impact of host identity on the microbiome of a trematode parasite

**DOI:** 10.1007/s00436-025-08486-0

**Published:** 2025-03-26

**Authors:** Xuhong Chai, Priscila M. Salloum, Robert Poulin

**Affiliations:** Department of Zoology, University of Otago, 340 Great King Street, Dunedin, 9016 New Zealand

**Keywords:** Host identity, Trematode microbiome, *Maritrema poulini*, Trematode parasite, Microbiome intraspecific variation, Alpha and beta diversity

## Abstract

Animal microbiomes have been recognized to have significant impact on animal behaviour, development, and health. For parasites, one of the sources of their associated microbes is likely their hosts, making host identity a potential factor explaining variation of microbial communities among conspecific parasites. Using the host-parasite system including the amphipod *Paracalliope fluviatilis*, and isopod *Austridotea annectens*, and their common trematode parasite *Maritrema poulini*, we hypothesized that host identity has an impact on the diversity and community composition of the microbiomes of *M. poulini* parasites. We focused on the bacterial component of the trematodes’ microbiomes. We investigated the effect of host identity under both intraspecific (among individual isopods) and interspecific (between amphipods and isopods) host levels and predicted that the effect of host identity can be detected under both host hierarchical levels. We found an effect of host identity on both the diversity and community composition of parasite microbiomes between conspecific isopod hosts, while host identity only had an impact on microbiome community composition when comparing parasites infecting amphipod versus isopod hosts. Our study results show that host identity impacts both parasite microbiome composition and taxonomic diversity, providing indirect evidence that the assembly of parasite microbiomes is not merely random, and that horizontal transmission from the host to the parasite might play a key role in shaping parasite microbiomes.

**Supplementary Information:**

The online version contains supplementary material available at 10.1007/s00436-025-08486-0.

## Introduction

Mounting evidence now shows that animal microbiomes impact multiple aspects of an animal’s life. For instance, microbiomes play critical roles in maintaining a healthy digestion system (Ley 2010), regulating the immune system’s performance (Schnupf et al. 2013), and shaping animal behaviour (Ezenwa et al. 2012). Therefore, variation in diversity and composition of microbiomes among individuals can account for intraspecific variation in phenotype and fitness. In addition, a portion of every animal’s microbiome must be inherited across generations (vertical transmission) (Greiman et al. 2013). However, many microbes are also acquired from the external environment (horizontal transmission), and this is likely an important source of inter-individual variation in microbiome composition (Salloum et al. 2023a).

Parasites, too, have microbiomes distinct from those of their hosts or the external environment (Dheilly et al. 2017; Jorge et al. 2022a). The composition of a parasite’s microbiome can also be associated with its phenotype; for example, the developmental rate or transmission route of certain parasites is associated with the diversity of their microbiome (Jorge et al. 2022b; Salloum et al. 2023b); parasites harbouring certain viral or bacterial taxa are either better at infecting their host (Martinson et al. 2020) or are possibly capable of greater manipulation of their host’s behaviour (Dheilly et al. 2015a) than their conspecifics harbouring different microbes (assuming those are not obligate microbes for the parasites). For parasites, the host represents the most immediate external environment, and thus the most likely source of horizontally acquired microbes. Parasites do have a ‘core’ microbiome that differs from both that of their hosts and that of the outside environment, however they do share many microbes with their hosts which are likely acquired horizontally (Jorge et al. 2020; Sheehy et al. 2022). Consequently, host identity probably accounts for much of the variation in microbiome composition among conspecifics parasites. Two individual parasites can either infect the same host individual, two distinct host individuals of the same species, or even two host individuals of different species, resulting in increasingly different source pools of microbes available for horizontal acquisition.

The aim of this study is to test whether host identity has an impact on the alpha (diversity) and beta diversity (community composition) of parasite microbiomes. The host-parasite system that was used in this study consisted of the amphipod *Paracalliope fluviatilis*, and isopod *Austridotea annectens*, and their common trematode parasite *Maritrema poulini*. The trematode *M. poulini* (Microphallidae) is a trophically transmitted parasite, which uses the New Zealand mud snail, *Potamopyrgus antipodarum*, as first intermediate host; isopods (*Austridotea annectens* or *A. lacustris*) or amphipods (*Paracorophium excavatum* or *Paracalliope fluviatilis*) as second intermediate hosts; and waterfowl as definitive hosts. Inside its isopod and amphipod hosts, *M. poulini* encysts as a metacercaria, entering a resting stage waiting for its crustacean host to be ingested by its definitive waterfowl host (Presswell et al. 2014). After infecting a suitable avian host, the trematode will reach adulthood and reproduce, and its eggs will be passed out in host faeces into the environment, where the entire life cycle will start again. After infecting an isopod or amphipod host, the cercaria loses its tail and eventually becomes encysted within the host, a process which could take days and gives plenty of time for possible horizontal acquisition of host microbes by parasites. The prevalence of *M. poulini* in its crustacean hosts at our study site is nearly 100% in *A. annectens* and a little less than 50% in *P. fluviatilis* (Presswell et al. 2014).

We hypothesize that the host identity has an impact on the alpha and beta diversity of the microbiomes of *M. poulini* parasites. We focus on the bacterial component of the trematodes’ microbiomes. Specifically, we predicted that: (i) metacercariae infecting the same individual host have more similar microbiomes than metacercariae infecting distinct conspecific host individuals; (ii) the similarity in microbiome composition of metacercariae infecting crustaceans of the same species is greater than that of metacercariae infecting crustaceans belonging to different species. In this system, the crustaceans used as second intermediate hosts belong to different orders, and thus likely expose the trematode’s metacercariae to different living conditions and different source pools of microbes for potential horizontal acquisition. We test the above predictions with parasites obtained from hosts collected at the same time from the exact same location, to eliminate as much as possible any effect of the external environment.

## Materials and methods

### Amphipods and isopods collection and dissection

In March 2023, *Paracalliope fluviatilis* amphipods and *Austridotea annectens* isopods were collected in Lake Waihola (46°01′S, 170°05′E), South Island, New Zealand. Prior to fieldwork, all sampling equipment and containers were sterilized with TriGene Advance Disinfectant (1:100 dilution, soaking 15 min). All amphipods and isopods were collected by scraping a dip net (500 μm nylon mesh) against the lake floor, near the shore in water < 1 m deep, following Daniels et al. (2013). Amphipods and isopods were separated from the lake bottom gravel using a 5mm mesh sieve and then transported to the laboratory in 5L plastic containers filled with lake water. Two environmental samples (lake water) were also collected at the site with cotton swabs, chilled with dry ice during transportation and stored at −70°C. Lake water was collected, and used to keep the amphipods and isopods alive in the laboratory. For the duration of dissections (14 days), animals were kept in sterilized plastic containers fitted with a sterilized bubbler. To sample the immediate outside environment of the animals (lake water in plastic containers), two samples were collected using cotton swabs and stored at −70°C until further processing.

Amphipods and isopods were dissected under a laminar flow hood. Prior to dissection, all equipment was sterilized by 15 min UV exposure. To avoid cross contamination between each dissected animal, all dissecting tools were cleaned and sterilized with TriGene Advance Disinfectant (1:100 dilution) and then rinsed with Milli-q water between consecutive dissections. *Maritrema poulini* metacercariae (as well as the whole-body tissue of infected and uninfected amphipods and hemolymph of isopod hosts) were extracted from amphipods and isopods and stored individually. To mechanically remove the microbes on the external surface of hosts and parasites, each of them was washed by being pipetted up and down in sterilized culture plate wells filled with heat-sterilized PBS solution. Washing in PBS solution to mechanically remove the surface microbes of parasites is a practice used previously (Jorge et al. 2020; Salloum et al. 2023a, b, c), and considered a suitable solution given the size of the parasites. All samples collected, along with 100 µl of the PBS solution (negative control), were stored separately in LoBind microcentrifuge Eppendorf tubes, which were then stored in a −70°C freezer until further processing.

### DNA extraction, library preparation, and bioinformatics

DNA extraction from all *M. poulini* samples collected was conducted with ZymoBIOMICS DNA Microprep Kit using the manufacturer’s protocol, with minor modification to break cells that were difficult to lyse (20 min bead beating with maximum speed of the bead beater), following Marotz et al. (2017). To enable quality checks and identification of potential contamination, DNA was also extracted from the ZymoBIOMICS microbial community standards sample (MCS), one negative control for PBS solution (100 µl PBS), 4 environmental cotton swab samples (two of the lake water, two of the containers’ water), as well as negative controls for all DNA extractions (6 DNA negative controls in total, which contained the DNA extraction kit reagents only and were pooled for sequencing).

The library preparation method was modified from the protocol in Jorge et al. (2020). The V4 hypervariable region of the prokaryotic bacterial 16S SSU rRNA gene was targeted using the universal bacterial forward primer 515F and reverse primer 806R (Apprill et al. 2015; Parada et al. 2016). In addition to all samples and controls mentioned above, two PCR negative controls (contained PCR reagents only) were included to enable identification of contaminants. Triplicate reactions were performed, each in a 25 µl mix composed of 12.5 µl Nuclease-Free Water (NORGEN BIOTEK CORP.), 5 µl MyTaq Red 5 × reaction buffer (Bioline), 0.5 µl MyTaq™ Taq DNA polymerase (Bioline), 2 µl of each Forward and Reverse primers, and 3 µl DNA template. The same PCR conditions as in Jorge et al. (2020) were used in this study, which consisted of 3 min at 95 °C for initial denaturation, followed by 35 cycles of 45s at 95 °C, 60s at 50 °C, and 90s at 72 °C, then 10 min at 72 °C for final extension. Triplicate libraries of each sample were pooled and run on a 2% agarose gel to check for successful amplifications. The amplicons were purified using Mag-Bind® TotalPure NGS Kit (Omega Bio-Tek) at a ratio of 0.8:1 solution to PCR product. DNA concentration of each sample was then quantified with Invitrogen™ Quant-i™ 1X dsDNA HS Assay Kit. Each amplicon library was manually normalized and equal volumes of amplicons were combined into a single tube to construct the final libraries pool. The pooled library was sequenced with an Illumina MiSeq platform and v3 reagent cartridge (250 bp, paired-end) at the Otago Genomics & Bioinformatics Facility. Raw sequencing reads were deposited in SRA (BioProject PRJNA1201596).

Sequence quality check on the demultiplexed paired-end raw sequences was carried out with FastQC v0.11.9 (Andrews 2010). The sequencing files were then imported into Qiime 2 v2023.5 (Bolyen et al. 2019), and the adaptors, overrepresented sequences and primers were removed using the *cutadapt* plug-in (Martin 2011), with 0 error rate and minimum length of 120 bp. Both forward and reverse sequences were trimmed by 13 bp, removing bases with lower quality at the start and end of the reads. Sequence errors were removed and similar sequences were combined using the denoising *dada 2* plug-in implemented in Qiime2 (Callahan et al. 2016). An amplicon specific naïve Bayes classifier was trained on the data of this study to later be used to assign taxonomy to the sequences. The classifier was trained by carrying out the following procedures: the SILVA database SSU Ref NR99 version 138.1 (Quast et al. 2013) was downloaded into Qiime 2, with low-quality sequences in the dataset being removed with *cull-seqs* plugin default parameters; sequences in the dataset were then filtered based on: sequences minimum length of 900 bp for Archaea, 1200 bp for Bacteria and 1400 bp for Eukaryota; sequences were de-replicated with *uniq* mode; the amplicon region from the reference database was extracted by using the forward primer sequence GTGYCAGCMGCCGCGGTAA and reverse primer sequence GGACTACNVGGGTWTCTAAT. Taxonomy was then assigned with this trained SILVA classifier using the Qiime2 *feature-classifier* plugin with *sklearn* mode. Feature tables (containing the frequency of each amplicon sequence variants–ASVs observed in each sample) were filtered to remove features that were without a phylum assignment or assigned to mitochondria, chloroplasts, or eukaryotes. The R package Decontam v1.22.0 (Davis et al. 2018) was used to identify contaminants based on both the prevalence method (cut off threshold: 0.5 probability) and frequency method (cut off threshold: 0.1 probability). To assess the performance of DNA extraction and PCR, the expected and observed composition of MCS were compared. All features from the MCS sample were then removed from the filtered data, as they would have most likely resulted from cross contamination of the MCS sample to the actual host and parasite samples. The final feature table was created by samples with a minimum of 500 features (based on the rarefaction curves generated, Fig. S5) and features with a minimum frequency of 2 samples, thus excluding 54 samples and 286 features from the dataset.

## Data analysis

Bioinformatically filtered datasets were imported into R studio v2024.04.2 (Posit team 2024) with the file2meco R package v0.6.0 (Liu et al. 2022), using the qiime2meco function. Most statistical analyses were performed with the R package microeco v0.11.0 (Liu et al. 2021). In addition, the R package phyloseq v1.46.0 (McMurdie and Holmes 2013) was used to agglomerate ASVs at different taxonomic levels with the function tax_glom, and ggplot2 v3.4.4 (Wickham and Wickham 2016) was used to generate visualizations. The relative abundance of bacterial taxa in *M. poulini* metacercariae was visualized with a non-rarefied dataset, for parasites of different isopod hosts, and parasites of amphipod and isopod hosts (mean relative abundance among conspecifics). Relative abundance was visualized at phylum and family levels with bar plots and heatmaps. A Venn diagram at ASV level was used to visualize the shared and different microbial taxa between metacercariae sampled from amphipod and isopod hosts. The rarefied dataset (with 581 sequences in each sample) was used for analyzing both the microbial diversity (alpha diversity) and the microbial community composition (beta diversity) of parasites at phylum, family, and ASV level.

To detect the impact of host identity on the microbiome of *M. poulini*, both alpha and beta diversity for parasite microbiotas were compared at two host hierarchal levels: 1) comparison of the microbiotas of parasites sampled from the same isopod individual against microbiotas of parasites sampled from different isopod hosts; 2) comparison of the microbiotas of parasites sampled from isopod hosts against the microbiotas of parasites sampled from amphipod hosts.

In addition, comparisons between: 1) the microbiotas of amphipods and the microbiotas of metacercariae sampled from amphipods; and 2) the microbiotas of isopods and the microbiotas of metacercariae sampled from isopods, were conducted at ASV level with all hosts and parasites microbiome data points which passed the filtering process. These comparisons served to detect whether there was a difference between the microbiotas of hosts and parasites. They were carried out with both Venn diagrams (also including the environmental samples for comparison) and beta diversity contrasts of microbiotas between hosts and parasites.

### Alpha diversity

To test whether host identity influences the alpha diversity of *M. poulini* microbiota, observed richness (i.e., number of features), the Shannon Diversity index, Faith’s phylogenetic diversity (PD), and the Simpson diversity index were estimated. To compare the microbiota alpha diversity between parasites from the same isopods and parasites from different isopods, alpha diversity was calculated with the cal_alphadiv function in the microeco R package. Pairwise distances (i.e. differences) in microbiome alpha diversity values were determined for all parasite pairs and categorized as sampled from the same isopod host or sampled from different isopod hosts. An Unpaired Two-Samples Wilcoxon Test was performed to compare these distances in alpha diversity between *M. poulini* sampled from the same and different hosts. Not all microbiome data for *M. poulini* sampled from isopods passed the bioinformatics filtering, therefore, only the microbiome data from *M. poulini* in isopods (*n* = 8) that had more than one parasite (2–3 individuals per isopod) passed the filtering were used to generate the pairwise dataset. This served to minimize the unbalanced number of data points between these two groups (i.e., *M. poulini* originating from the same individual host versus from different host individuals). Wilcoxon Rank Sum Tests (cal_diff function) were carried out to compare the microbiota alpha diversity (using same metrics as above) between parasites sampled from amphipod and isopod hosts.

### Beta diversity

To test whether host identity influences the beta diversity of *M. poulini* microbiota, the beta diversity metrics Bray–Curtis, Jaccard, weighted Unifrac, and unweighted Unifrac, were assessed at phylum, family, and ASV level. PerMANOVA (cal_manova function, 999 permutations) was carried out to compare the parasite microbiome under the two host hierarchies mentioned above (metacercariae from the same versus different individual isopod hosts, metacercariae from amphipod versus isopod hosts), and PCoA, box plots, and clustering plots were used for visualization. The PERMDISP test (Anderson 2001)(cal_betadisper function of the microeco R package) was implemented to test the multivariate homogeneity of the groups’ dispersions for the PerMANOVA test.

In addition, a PerMANOVA test was also used for comparing the beta diversity of: 1) amphipods microbiotas and the microbiotas of metacercariae sampled from amphipods, and 2) isopods microbiotas and the microbiotas of metacercariae sampled from isopods. The beta diversity metrics mentioned above were used to compare ASV level data. PCoA plots were used for visualization, and the PERMDISP test was also conducted.

### Differential abundance tests

To test whether the abundance of particular bacterial taxa differs significantly between samples, non-rarefied data were used for tests of differential abundance among the microbial communities of *M. poulini* sampled from amphipods versus isopod hosts. The methods used for differential abundance tests were ALDEx2 (here ALDEx2_t—Welch’s test method was used, as implemented in the cal_diff function of the microeco package) (Fernandes et al. 2014) and the corncob v0.3.0 R package (Martin et al. 2022), both of which are recommended by benchmarking studies (Nearing et al. 2022; Yang and Chen 2022). Tests for differential abundance were conducted with ASV data and taxa that showed significant differences were identified at phylum and family level. For data analysis, script, metadata, and filtered data are available from FigShare (Chai 2024 [dataset]).

## Results

In this study, the prevalence of *M. poulini* in amphipods was 46%, while in isopods it was 100%. In total, 14 metacercariae were obtained from 14 amphipods and 29 metacercariae were extracted from 14 isopods (2 per isopod, and 3 from one isopod). The whole-body tissue of these 14 infected amphipods and 14 uninfected amphipods, as well as the hemolymph of these 14 isopod hosts were also collected. Sequencing results for some samples (mostly hosts) were below the expected quality and these samples were excluded from downstream analyses (Table S1). The filtered dataset consisted of 40 samples and 1036 ASVs, ranging in coverage from 581 to 545,536 (mean = 17,493; SD = 85,747). Of these, one was an environmental sample (36 ASVs, coverage 984), six samples were amphipod (5 uninfected individuals, 1 infected individual) samples (54 ASVs, mean coverage 2,963, SD = 1,336), four samples were isopod hosts (51 ASVs, mean coverage 1,640, SD = 771), and 29 samples were parasites (961 ASVs, mean average 23,256, SD = 100,576). Of the latter, eight samples were *M.poulini* samples extracted from amphipod hosts (186 ASVs, mean coverage 1,799, SD = 1,908), and 21 samples were *M. poulini* samples extracted from isopod hosts (823 ASVs, mean average 31,429, SD = 117,924). The results for the Mock Community Standard quality control analyses showed that the observed and expected abundance for class level and above were highly accurate, with decreasing accuracy below order level (note that the rate of observed to expected taxa remains close to 1 for lower taxonomy levels) (Fig. S1).

Both Venn diagrams and PerMANOVA tests showed that the microbiotas of amphipods and isopods were overall different from the microbiotas of *M. poulini* metacercariae, and that there were very few shared ASVs between the environmental sample and the host and parasite samples (Fig. S2). However, the PERMDISP tests showed that some of the PerMANOVA results violated the multivariate homogeneity of group dispersions (Table S5, S7).

### Microbiome of *M. poulini* sampled from isopod hosts

**Fig. 1:**
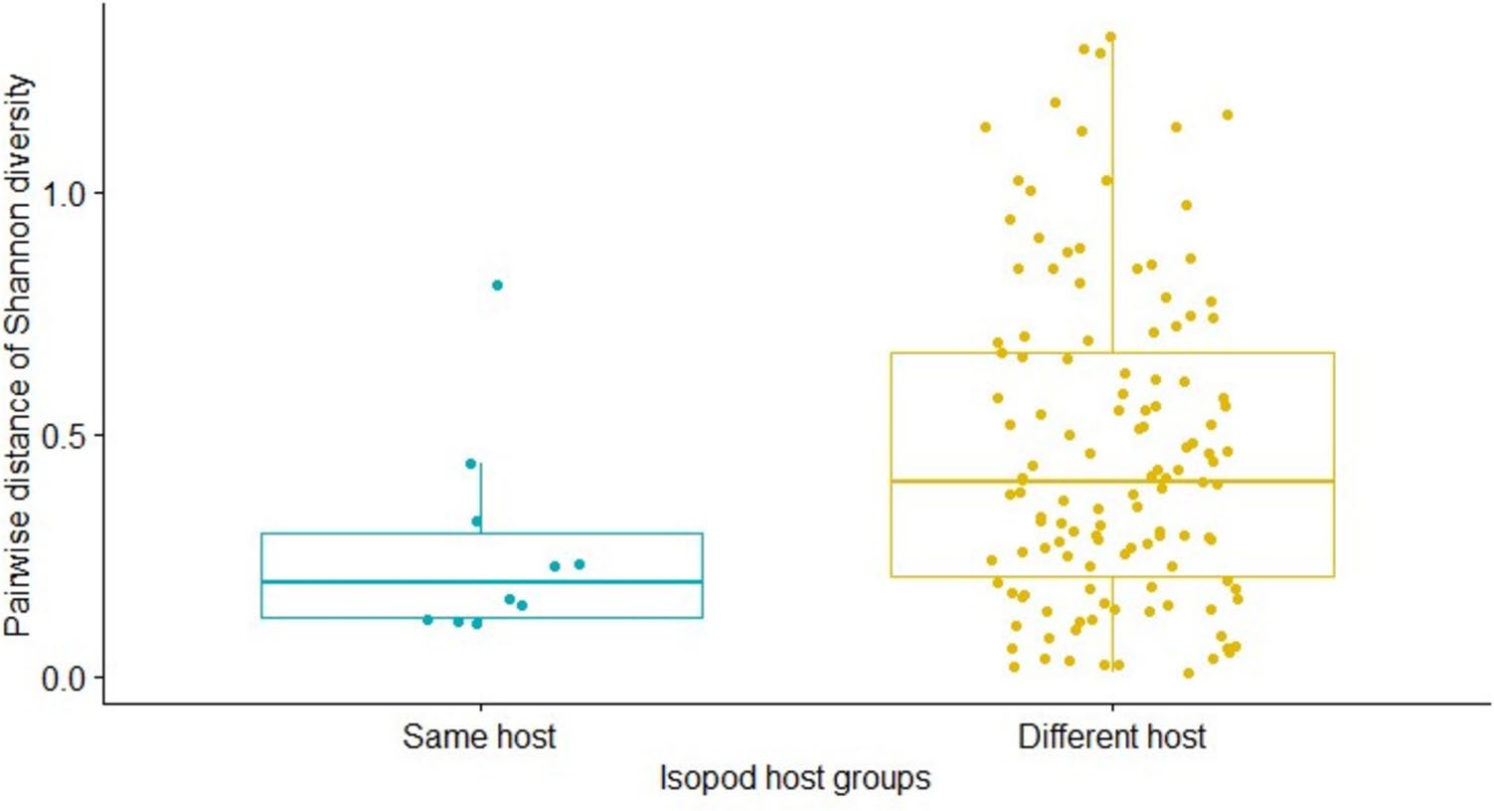
Pairwise distance of Shannon diversity between the microbiota of the trematode *Maritrema poulini* sampled from the same (in blue) and different (in yellow) individual isopod hosts. Each point represents a contrast between one pair of parasites, with all possible pairs included for both groups

**Fig. 2:**
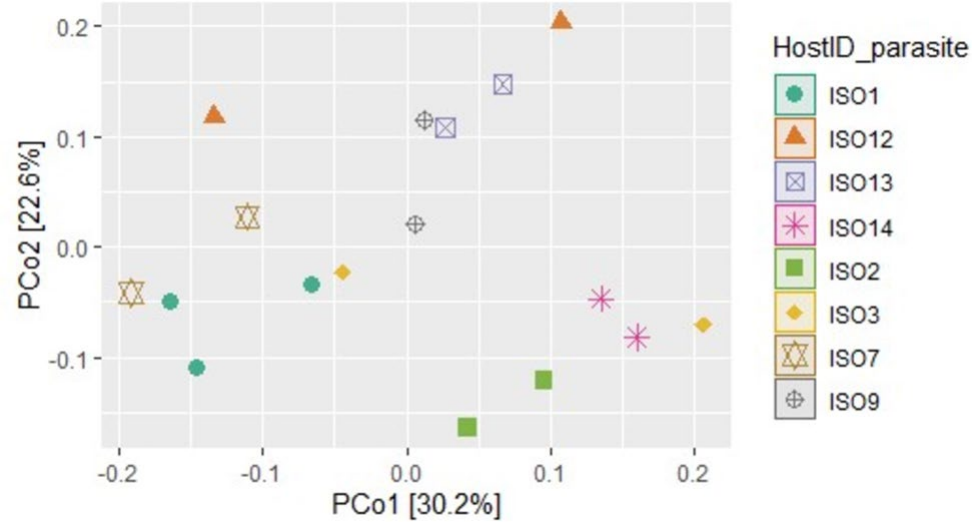
Beta diversity of the bacterial community of *Maritrema poulini* sampled from isopod hosts, comparing those sharing the same host (same symbol) with those obtained from different hosts (different symbols). The PCoA is based on – Weighted Unifrac distances at ASV level. ISO1 to ISO14 are the isopod hosts sampled for parasites

**Fig. 3:**
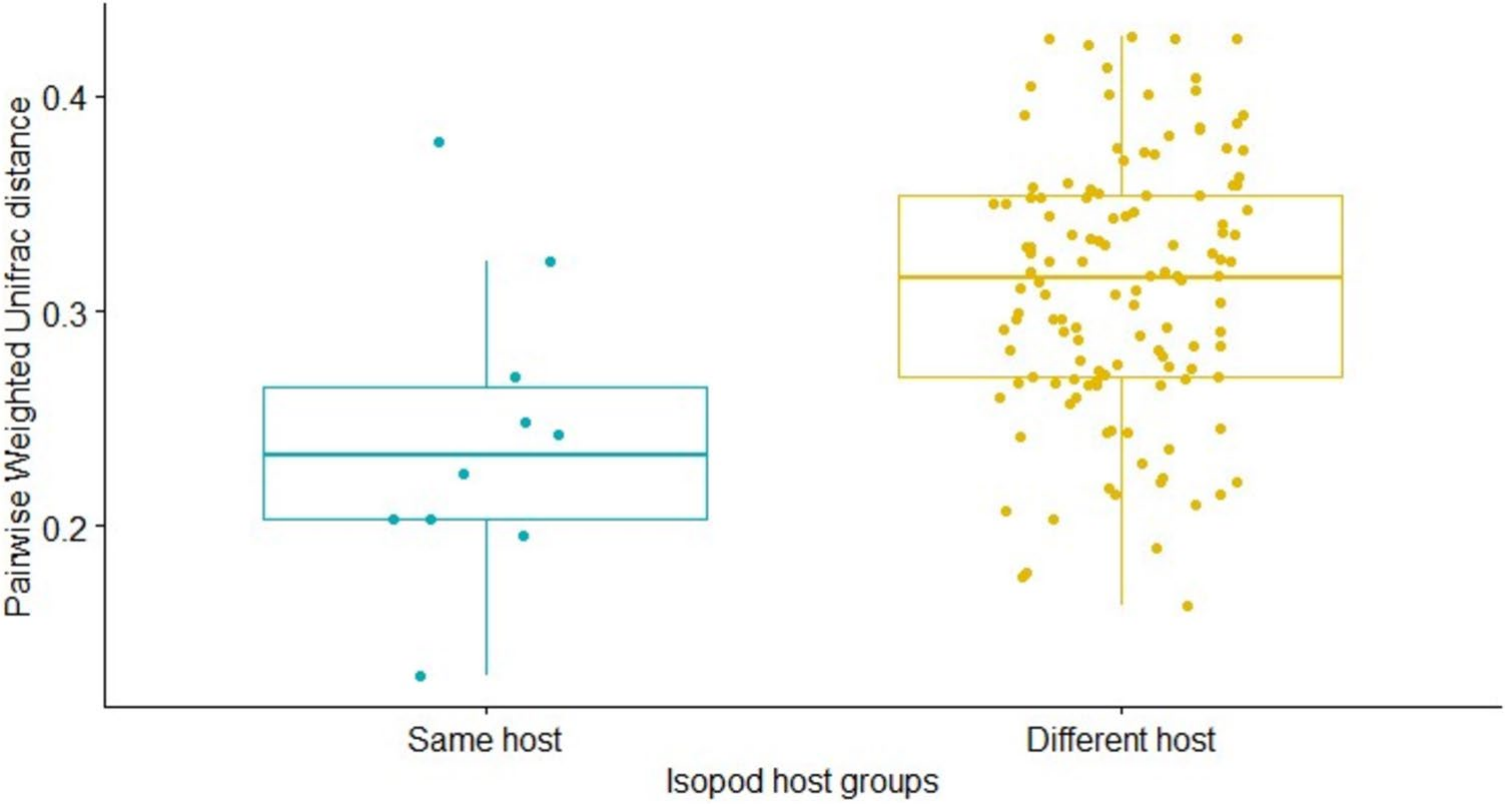
Pairwise distance of Weighted Unifrac between the microbiota of the trematode *Maritrema poulini* sampled from the same (in blue) and different (in yellow) individual isopod hosts. Each point represents a contrast between one pair of parasites, with all possible pairs included for both groups

The unpaired two-samples Wilcoxon test showed that, at both family and ASV levels (but not at phylum level), the differences in microbial diversity were higher between *M. poulini* parasites infecting different isopod host individuals than between parasites of the same isopod host, for both Shannon diversity (family level: W = 875, *p* = 0.041) and Simpson metrics (family: W = 898, *p* = 0.026; ASV: W = 931, *p* = 0.012) (Fig. 1). However, this was not the case for Observed Richness and Faith’s PD, at all three tested taxonomic levels. The PerMANOVA test showed that the differences in microbiome composition were higher between *M. poulini* occurring in different isopod individuals than between parasites harboured by the same isopod individual, at all three taxonomic levels tested (except Bray–Curtis and weighted Unifrac metrics at phylum level) (Figs. 2, 3, Table S2). Indeed, points representing the microbiomes of metacercariae from the same individual isopod tend to cluster together in the PCoA plot (Fig. 2). However, the betadisper test showed that almost all of the perMANOVA results at phylum and family levels violated the multivariate homogeneity of groups’ dispersions, but not at the ASV level (Table S2).

### Microbiome of *M. poulini* from amphipod versus isopod hosts

**Fig. 4:**
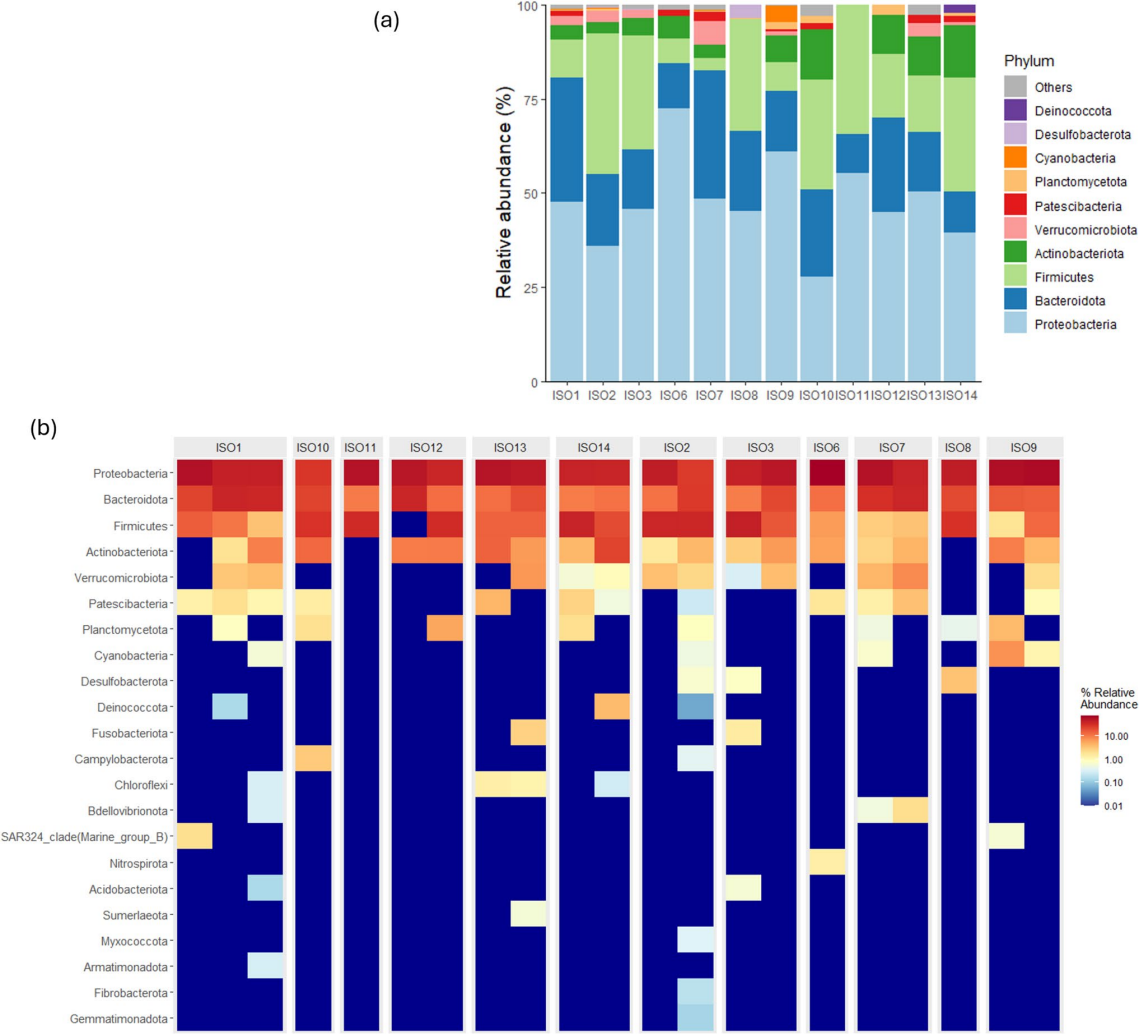
Relative abundance of bacterial phyla within the microbiomes of *Maritrema poulini* metacercariae from isopod hosts. **a** Bar plots of the relative abundance of bacterial phyla, with metacercariae from the same individual isopod grouped for analysis; **b** Heatmaps of relative abundance, with each column representing the microbiome of a separate metacercaria. ISO1 to ISO14 are the isopod hosts sampled for parasites, and each column under an isopod host represents one individual metacercaria

**Fig. 5:**
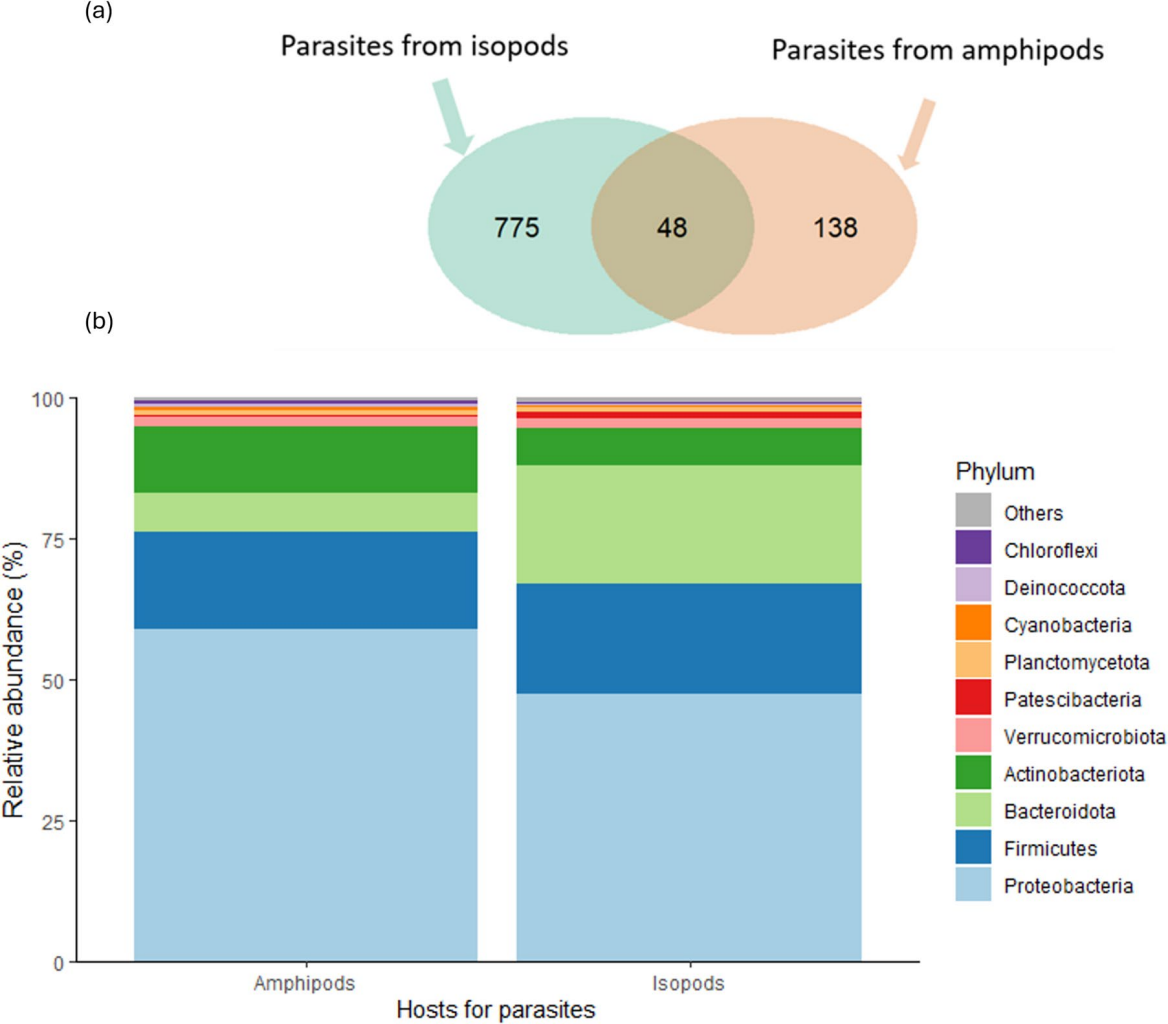
Comparison of microbiome composition in *Maritrema poulini* metacercariae sampled from amphipod and isopod hosts. **a** Venn diagram based on number of ASVs unique or shared between the two groups: *M. poulini* sampled from amphipod and isopod hosts; **b** Bar plots of the mean relative abundance of bacterial phyla in the microbiomes of metacercariae from amphipod and isopod hosts

**Fig. 6:**
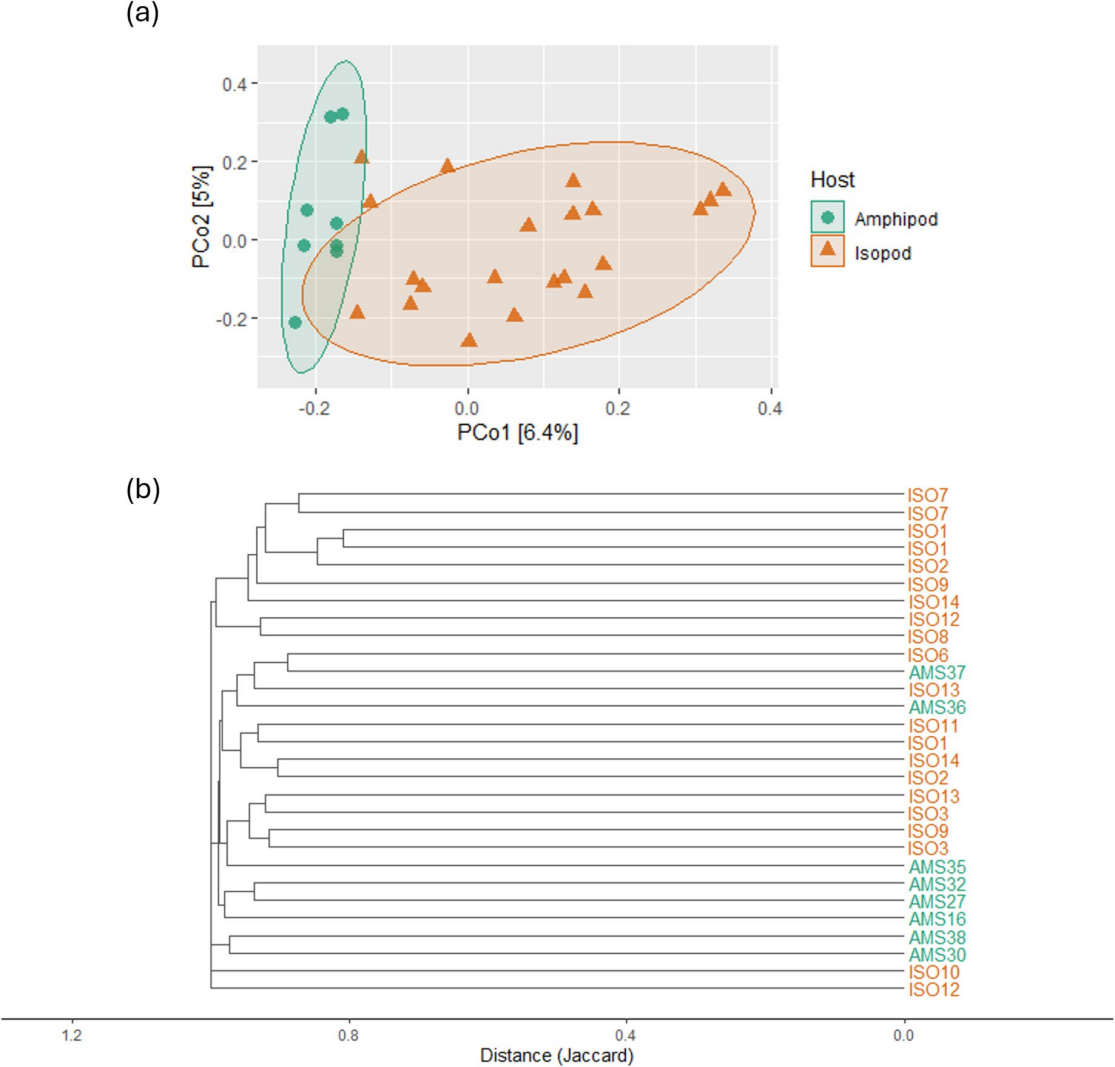
Comparison of beta diversity (based on ASV level data with Jaccard distance) of microbiotas of *Maritrema poulini* metacercariae infecting amphipod and isopod hosts. **a** PCoA plot; **b** clustering plot. ISO1 to ISO14 are the isopod hosts sampled for parasites, whereas AMS16 to AMS38 are the amphipods sampled for parasites

Based on the bar plots of relative abundance, the most prevalent bacterial phyla in *M. poulini* microbiomes from both amphipods and isopods were Proteobacteria, followed by Bacteroidota, Firmicutes, and Actinobacteriota (Fig. 4, Fig. S3). At family level, the most prevalent taxa were Rhodobacteraceae, Comamonadaceae, and Pseudomonadaceae (Fig. S3, S4). There were 48 shared ASVs between the microbiota of *M. poulini* sampled from amphipod and isopod hosts (Fig. 5a). *Maritrema poulini* infecting isopod hosts had more unique ASVs than those infecting amphipods (775 vs 138 ASVs, Fig. 5a). Wilcoxon Rank Sum Tests showed there was no difference in alpha diversity between the microbiome of *M. poulini* metacercariae infecting amphipod and isopod hosts at phylum, family, and ASV levels. However, in terms of beta diversity, there were significant differences in the composition of *M. poulini* metacercariae between those infecting amphipods and isopods at phylum (except Jaccard and unweighted Unifrac), family, and ASV levels (Table S3, Fig. 6). Both ALDEx2_t (p.adjust = 0.006) and corncob (*p* < 0.001) differential abundance tests (along with mean relative abundance bar plots, Fig. 5b) showed that the phylum Bacteroidota achieved higher relative abundance in the microbiomes of *M. poulini* sampled from isopods than in those from amphipods (Fig. S5). However, no significant difference in relative abundance was found between the microbiomes of metacercariae infecting the two different host species for any bacterial taxa at family level.

## Discussion

Intraspecific variation of microbiome communities (alpha diversity and/or community composition) among conspecifics, even if collected from the same locality, has been observed in many helminths (Hahn et al. 2022; Jorge et al. 2022b). However, the factors that result in this variation remain unknown. To an endoparasite, the host is the most immediate external environment, making it possible for a parasite to acquire some of its host’s microbes. Hence, because different hosts themselves are unlikely to harbor identical microbiomes, host identity might play a role in producing intraspecific variation of parasite microbiome communities. Given the likely phenotypic and fitness impacts of microbial communities on the parasites harbouring them (Ezenwa et al. 2012), this variation may have important consequences. Here, we compared the microbiomes of *Maritrema poulini* trematodes across two host hierarchies: 1) between trematodes infecting conspecific isopod hosts; 2) between trematodes infecting different host species (amphipods and isopods). We found an effect of host identity for both the alpha diversity and community composition of parasite microbiotas between conspecific isopod hosts, while host identity only had an impact on microbiota community composition when comparing parasites infecting amphipod versus isopod hosts. However, low pairwise distance for the alpha diversity metrics between two parasite microbial communities cannot rule out the possibility that these two communities have completely different bacterial taxa. Nevertheless, this doesn’t limit the impact of our results, since the influence of host identity was identified throughout almost all beta diversity metrics at all three tested taxonomic levels (phylum, family, ASV).

Through horizontal transmission, parasites might obtain parts of their microbiome from a conspecific parasite sharing the same host, a condition which is likely to happen when there is a high intensity of conspecific parasites within same host individual (Salloum et al. 2023a). In this study, most of the infected amphipods contained only one metacercaria (few harboured 2–4 metacercariae), while each infected isopod individual harboured dozens of metacercariae, suggesting metacercariae within isopods are potentially exposed to more frequent parasite-parasite microbiome exchange events, as the crowding of metacercariae within the same isopod host might increase the chances of microbial exchange between metacercariae. This is one of the factors that might lead to higher similarity in both alpha and beta diversity of *M. poulini* microbiomes when sampled from within the same isopod host individual than from different isopod hosts.

Parasites can also acquire microbes from their hosts by horizontal transmission (Jorge et al. 2022a). In this study, both alpha and beta diversity of parasite microbiotas were less similar among parasites infecting different isopod hosts than among parasites infecting the same isopod host individual. In addition to parasite-parasite exchanges, this might reflect the potential influence of horizontal transfer of host microbes to parasites within each host individual. Here, even with though the hosts samples were not used due to low quality after sequencing, it was still possible to find common microbial taxa between *M. poulini* and hosts (around 5%, Figure S2 D). More taxa might be shared, but our low depth of sequencing for hosts samples precludes us from any meaningful inference in this regard. Two parasites sharing the same host would obviously be exposed to the same host microbes, and would thus acquire many of the same microbes and develop similar microbiomes. We also found that there was a difference of microbial community composition between parasites infecting amphipods and those infecting isopods. This pattern presumably results mainly from the horizontal transmission of microbes from hosts to parasites. Alternatively, parasites with different microbiomes could be more successful at infecting hosts of different species (Kaltz and Shykoff 1998). In other words, the parasites would have had different microbiomes prior to infection, and these microbiomes would be a factor pre-determining which hosts they end up infecting. This scenario seems less likely than the horizontal acquisition of microbes from the host post-infection.

The differential abundance tests comparing the microbiomes of parasites of amphipods versus those of isopods have revealed that the bacterial phylum Bacteroidota is more abundant in the parasites of isopods. This could indicate a greater propensity for this phylum to be acquired by horizontal transmission, in which case, presumably, bacteria of the phylum Bacteroidota occur at a much higher abundance in isopod haemolymph and are transferred horizontally at a higher rate from host to parasite than in amphipods. Differences in parasite microbiomes observed between amphipods and isopods might therefore reflect a difference between the amphipod microbiome and the isopod microbiome. The consequences of microbiome differences between isopods and amphipods may also explain infection patterns. For instance, the different prevalence achieved by *M. poulini* in amphipods (46%) and isopods (100%) may be due to differences between the two host species in body size, behaviour or some other trait. However, differences in microbiome composition and microbial abundance between the two hosts could also shape variation in their immune response to the same trematode (Dheilly et al. 2015b).

In addition, interactions between the two sympatric crustacean hosts may also play a role in determining their parasites microbiomes: the isopod might prey on the amphipod (Friesen et al. 2020), and therefore, obtain part of the amphipods’ microbes via predation (Tilg and Moschen 2015). This might explain some patterns: 1) the much higher abundance of ASVs in the microbiome of parasites infecting isopods than in those of parasites infecting amphipods (by ingesting amphipods, isopods may accumulate more abundant microbial taxa, which are represented by more ASVs); 2) the overlap in microbial ASVs between parasites sampled from amphipods and those found in isopods; 3) the fact that host identity only has an impact on the presence/absence of bacterial phyla for parasites sampled among conspecific isopod hosts, while only having an impact on the bacterial abundance for parasites sampled between different host species (as a food source of isopods, amphipods might mostly contribute microbes from the same phyla as those already occurring in isopods). However, the higher abundance of microbial ASVs in parasites of isopods could also be an artefact of the unequal numbers of samples (8 parasites sampled from amphipods, 21 parasites sampled from isopods) and unequal sequencing quality among samples in the final dataset (Fig. S6).

Furthermore, the weaker effect of host identity on microbiome community composition of parasites at phylum level (when compared to the impact of host identity at ASV level) might suggest that differences in bacterial taxa do not represent variation in function among microbiomes. Presumably microbial functions are conserved among closely related bacterial taxa, and thus the microbiomes of parasites infecting different host species may not differ greatly from a functional perspective (McFall-Ngai et al. 2013; Doolittle and Booth 2017). Also, the possibility that *M. poulini* harbour a certain proportion of microbes obtained from the external environment (lake water) cannot be ruled out, since the life cycle of *M. poulini* has a free-living cercarial stage, which swims freely in the lake prior to infecting crustacean hosts (Presswell et al. 2014). However, this study could not characterize the microbes in the external environment due to the low sequence quality of the environmental samples. Finally, there were 48 shared ASVs between the microbiota of *M. poulini* sampled from amphipod and isopod hosts, suggesting the existence of a core microbiome in *M. poulini*, as seen in other trematodes (Jorge et al. 2020). It will be interesting to investigate these ASVs in the future and whether they indeed occur at other life stages of the trematodes.

Overall, this study has shown that the microbiome of *Maritrema poulini* is affected by host identity, suggesting a potential impact of horizontal host-to parasite transmission on the microbiome of conspecific parasites. Similarly, Salloum et al. (2023c) found that the microbiome of another *Maritrema* trematode–*Maritrema novaezealandense* (from a marine system), has low stability of microbiotas among conspecific individuals and is presumably more susceptible to microbial horizontal transmission than other co-occurring trematodes. The impact of host identity on *M. poulini*’s microbiome supports the hypothesis of horizontal transmission in this parasite genus. Considering that *Maritrema* trematodes can be found in freshwater, brackish, and marine ecosystems (Presswell et al. 2014), whether their microbiome being strongly determined by horizontally transmitted microbes holds the key to their successful invasion of so many different ecosystems is unknown (van Oppen et al. 2018).

Even though the host microbiome could not be extensively characterized due to low number of samples passing bioinformatics filters (see results section), information on its diversity and/or composition would not change the conclusions of our study. Indeed, the comparison between the hosts (though with limited data points, see Table S4, S6) and parasites microbiotas indicates that the parasite microbiotas defined in this study are genuine parasite microbiotas rather than being the host microbiotas. The microbiota of hosts and trematode parasites were also found to be different in previous studies using the same methods we used (Jorge et al. 2020; Salloum et al. 2023a, b, c). We found that the variation in the microbial community of *M. poulini* metacercariae, sampled from the same population at the same time, could be explained by host identity (i.e. whether they came from the same host individual, from another conspecific individual, or from a different host species), providing indirect evidence that the assembly of parasite microbiomes is not merely random. Even for conspecific parasites, there is likely inter-individual variation in the degree of microbial exchange with their hosts, and potentially some exchange between co-infecting parasites within the same host. Several fitness related traits of parasites may be shaped by the composition of their microbiome, ranging from their infection success in the next host to their adult growth and fecundity (Dheilly et al. 2015b). However, there is also indication of a core microbiota, comprising bacterial taxa that could potentially be vertically transmitted among *M. poulini* and ultimately also have consequences for parasite fitness. Therefore, it is essential to further understand the host-parasite-microbiome dynamics to achieve a better understanding of the factors that have impact on the parasite microbiome, and of how this can influence the parasites’ fitness and impacts on its hosts.

## Supplementary Information

Below is the link to the electronic supplementary material.

Supplementary Material 1: The supplementary material for this article can be found on the Springer Nature Link platform alongside the article. (PDF 1.27 MB)

## Data Availability

Raw sequence reads are available in the SRA (BioProject PRJNA1201596, BioSamples SAMN45938543 to SAMN45938636). Script, filtered data, metadata, and gel electrophoresis images are available on FigShare (10.6084/m9.figshare.28039658).
