# Supplementary material for: Does the host matter? Testing the impact of host identity on the microbiome of a trematode parasite
Source: Parasitol Res. 2025 Mar 26;124(3):38. doi: 10.1007/s00436-025-08486-0 (PMC11947000; doi:10.1007/s00436-025-08486-0)
Supplement: Supplementary file 1 — Supplementary Material 1: The supplementary material for this article can be found on the Springer Nature Link platform alongside the article. (PDF 1.27 MB) [file 436_2025_8486_MOESM1_ESM.pdf]

Supplementary materials

Does the host matter? Testing the impact of host identity on the microbiome of a trematode parasite

Xuhong Chai*, Priscila M. Salloum, Robert Poulin

(a)

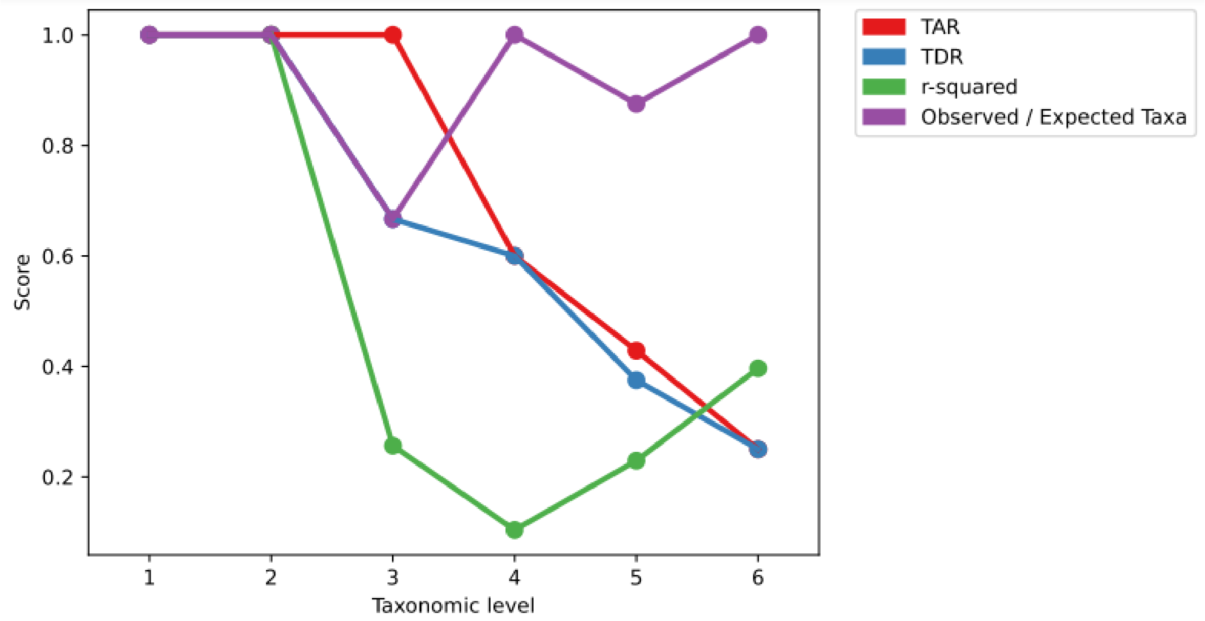

(b)

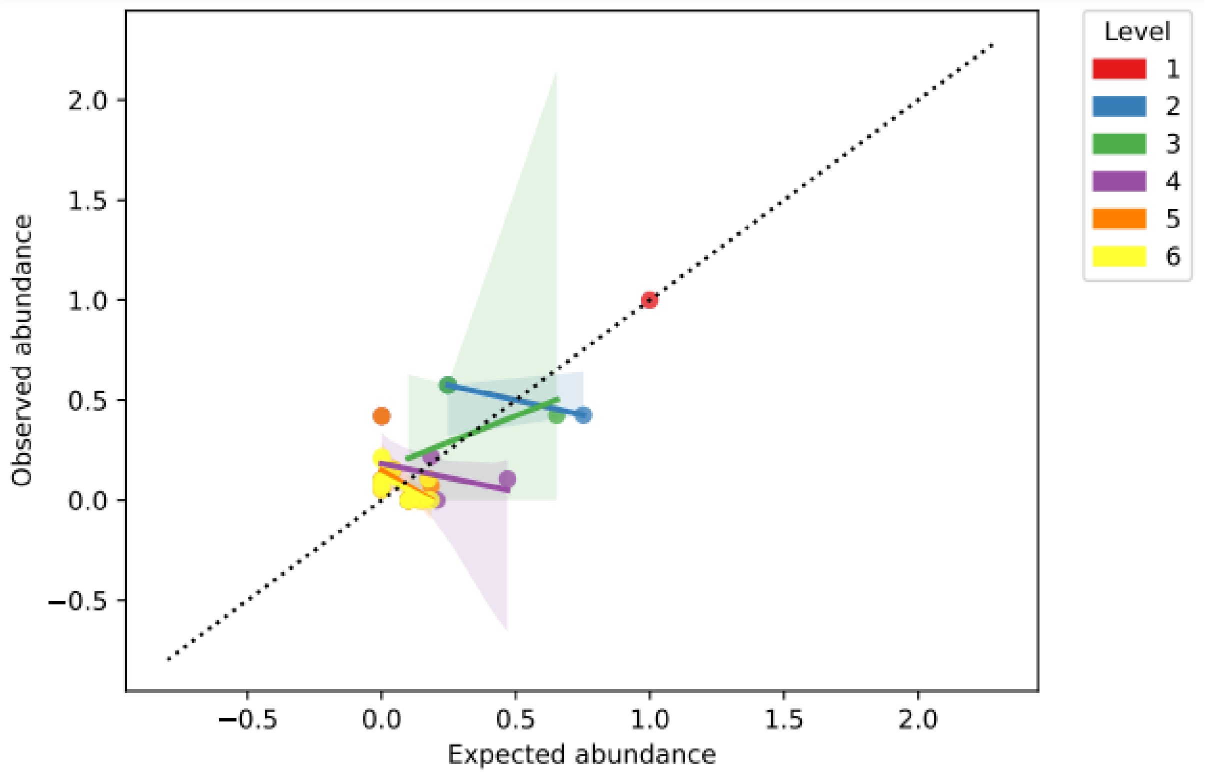

Figure S1. Mock community standard comparison (expected and observed), including negative controls. (a) Score plot accuracy per taxonomic level. Taxonomic accuracy score (TAR), taxonomic detection score (TDR), correlation coefficient (r-squared), and ratio of observed by expected taxa; (b) Correlation of observed and expected abundance for each taxonomic level. 1 = kingdom; 2 = phylum; 3 = class; 4 = order; 5 = family; 6 = genus.

(a)

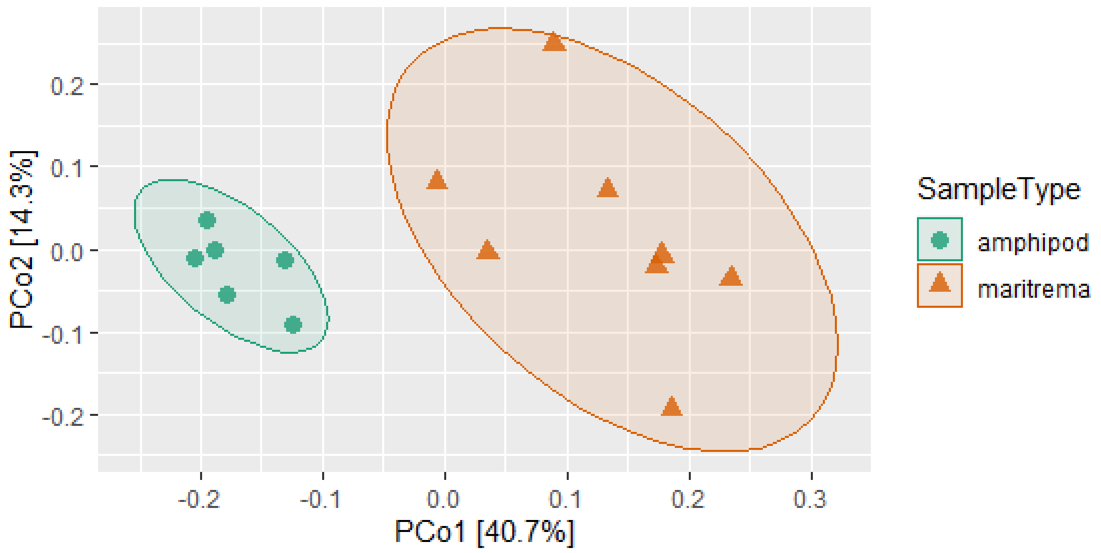
0.2
0.1
0.0
-0.1
-0.2
PCo2 [14.3%]
-0.2
-0.1
0.0
0.1
0.2
0.3
PCo1 [40.7%]
SampleType
amphipod
maritrema

(b)

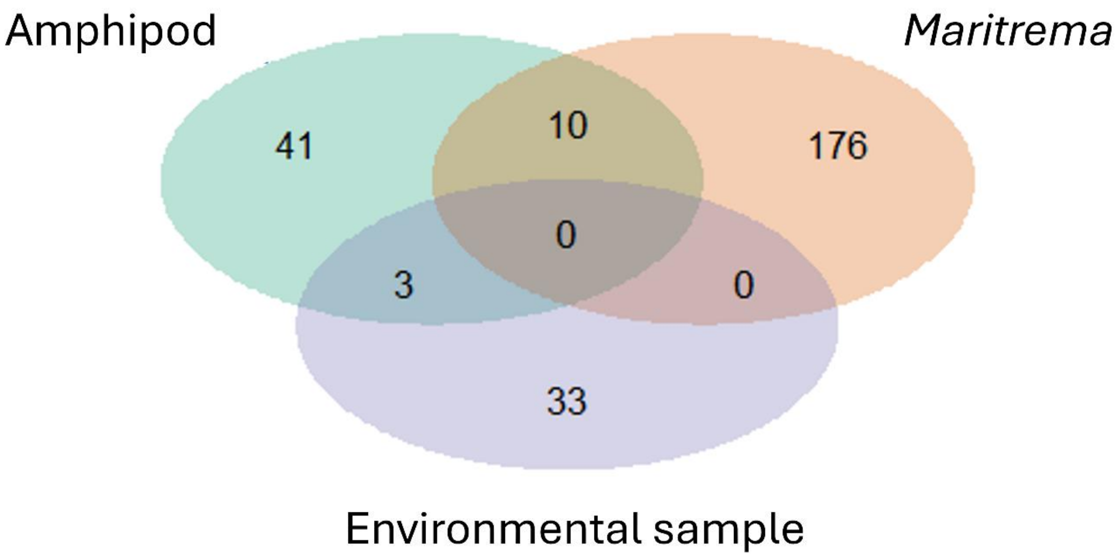
Amphipod
Maritrema
41
10
176
0
3
0
33
Environmental sample

(c)

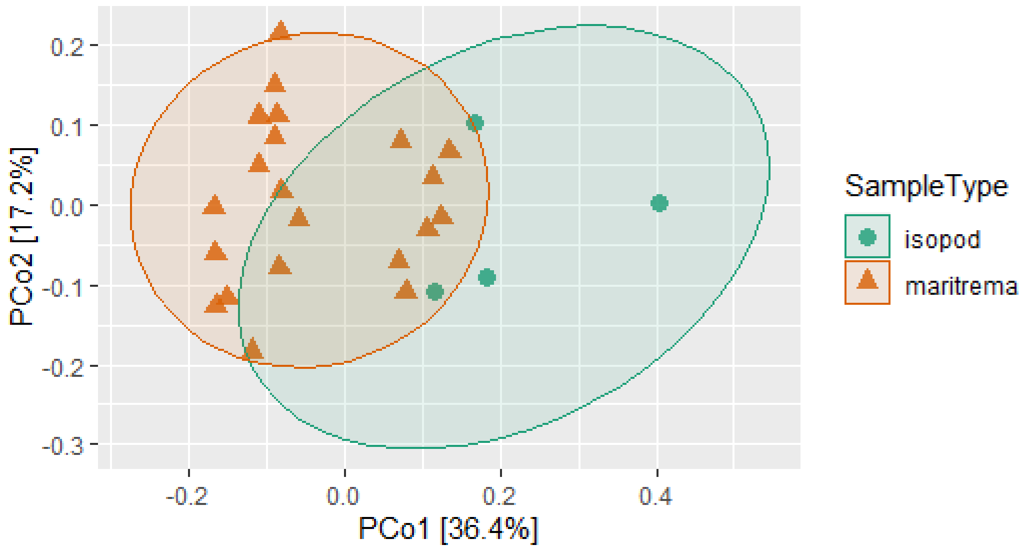

(d)

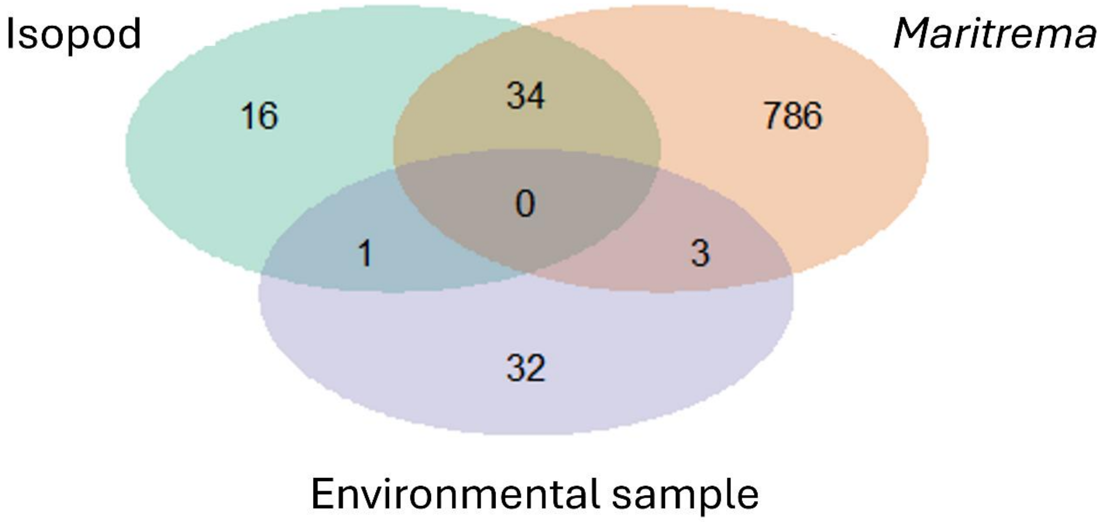

Figure S2. Comparisons of microbiome composition between hosts and parasites. PCoA plots for the comparison of beta diversity of the bacterial community between: (a) amphipods (n = 6) and *Maritrema poulini* metacercariae (n = 8) sampled from amphipods. Venn diagram based on number of ASVs unique or shared among the three groups: (b) amphipods (n = 4), *M. poulini* metacercariae (n = 8) sampled from amphipods, and environmental sample (n = 1, Lake water sample). PCoA plots for the comparison of beta diversity of the bacterial community between: (c) isopods (n = 4) and *M. poulini* metacercariae (n= 21) sampled from isopods. The PCoA is based on – Weighted Unifrac distances at ASV level. Venn diagram based on number of ASVs unique or shared among the three groups: (d) isopods (n = 4), *M. poulini* metacercariae (n = 21) sampled from isopods, and environmental sample (n = 1, Lake water sample).

(a)

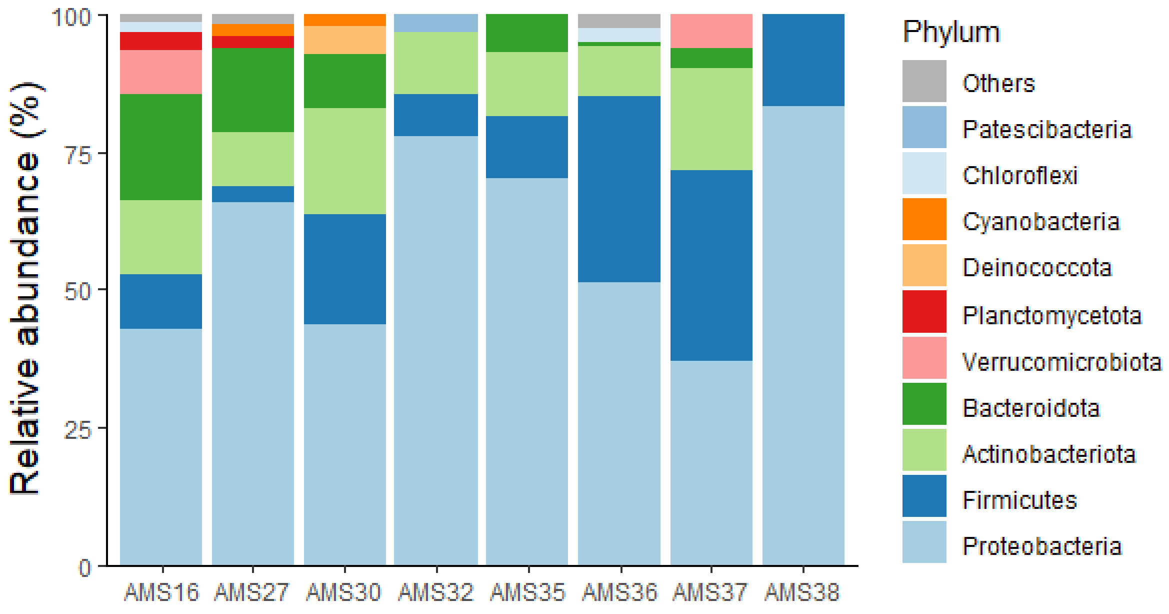
Relative abundance (%)
100
75
50
25
0
AMS16
AMS27
AMS30
AMS32
AMS35
AMS36
AMS37
AMS38
Phylum
Others
Patescibacteria
Chloroflexi
Cyanobacteria
Deinococcota
Planctomycetota
Verrucomicrobiota
Bacteroidota
Actinobacteriota
Firmicutes
Proteobacteria

(b)

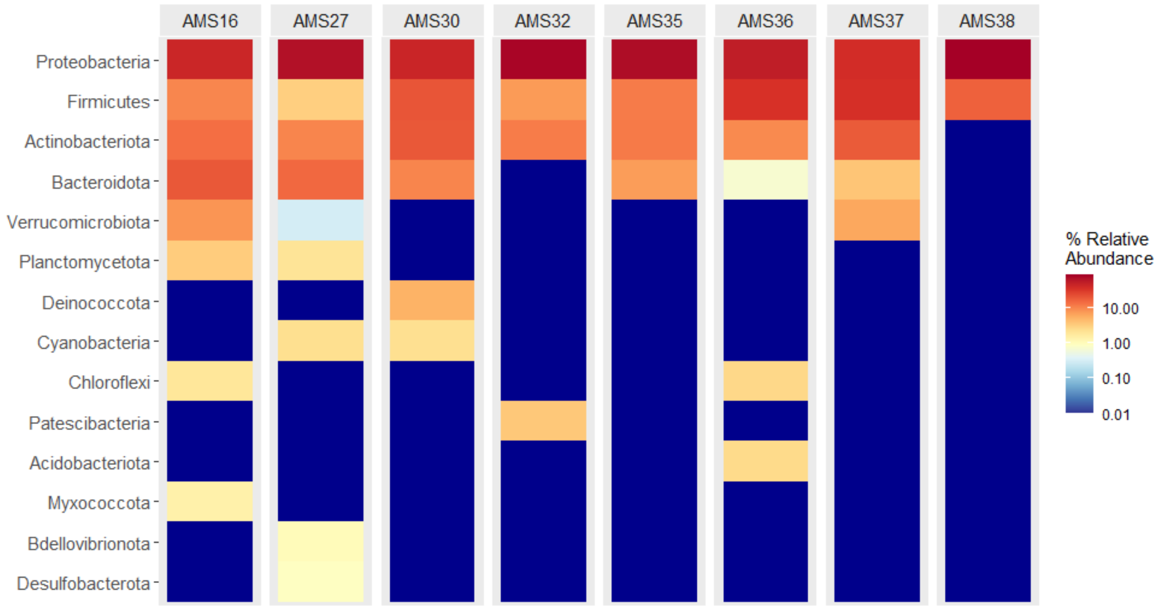
AMS16
AMS27
AMS30
AMS32
AMS35
AMS36
AMS37
AMS38
Proteobacteria
Firmicutes
Actinobacteriota
Bacteroidota
Verrucomicrobiota
Planctomycetota
Deinococcota
Cyanobacteria
Chloroflexi
Patescibacteria
Acidobacteriota
Myxococcota
Bdellovibrionota
Desulfobacterota
% Relative Abundance
10.00
1.00
0.10
0.01

(c)

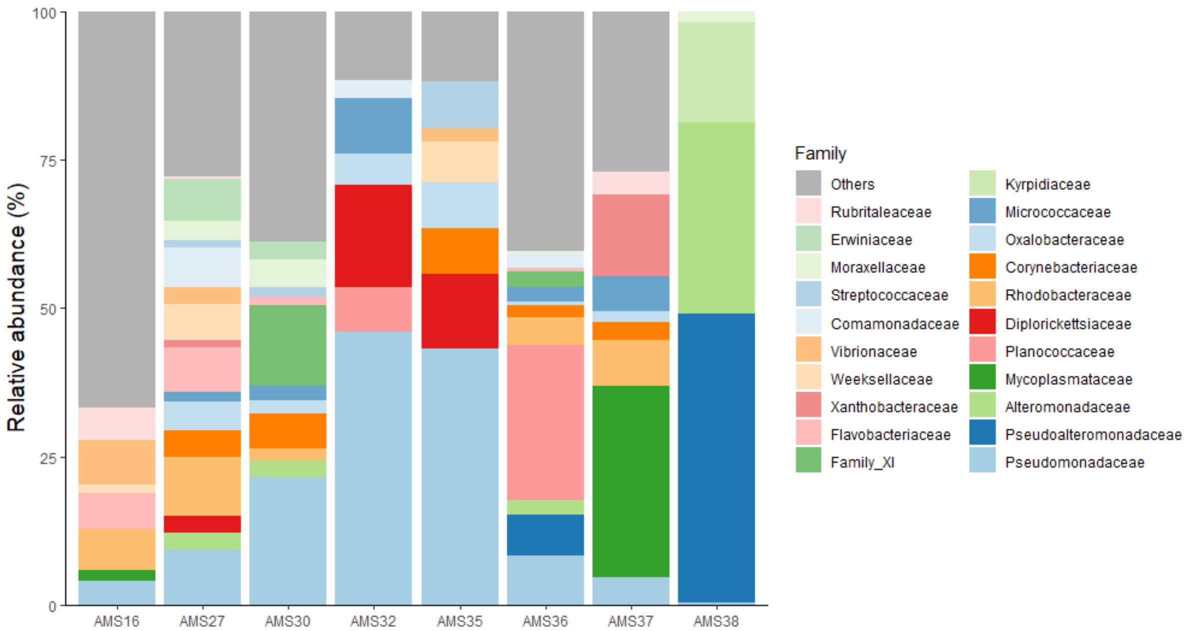

(d)

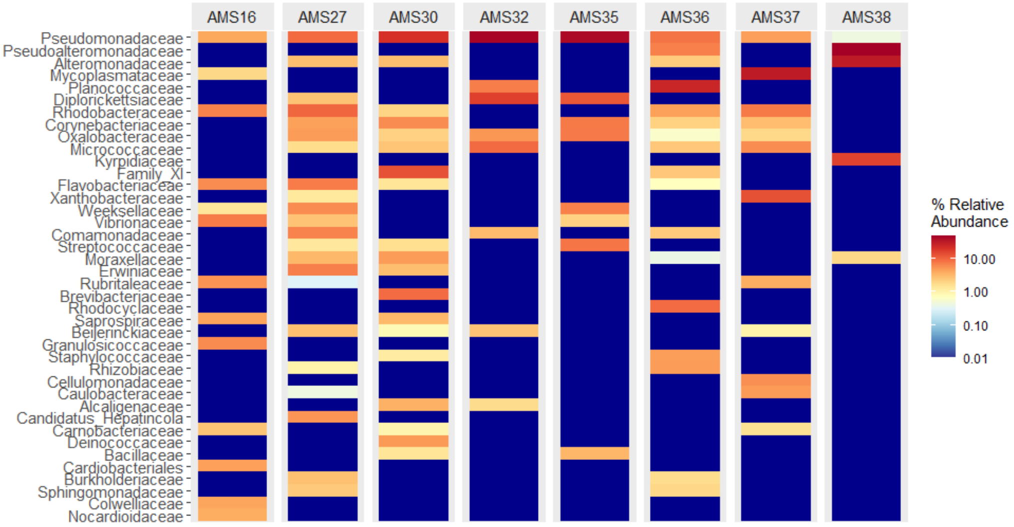

Figure S3. Relative abundance of microbiome (bacterial taxa) of *Maritrema poulini* sampled from amphipod hosts at (a-b) phylum and (c-d) family level. (a)(c) Relative abundance bar plots at (a) Phylum and (c) family level, x axis is each amphipod host; each bar plot represents mean relative abundance of bacteria in all *M. poulini* individuals from the same amphipod host; (b)(d) Relative abundance heatmaps at (b) phylum and (d) family level; each column represents relative abundance of bacteria in each *M. poulini* sample.

(a)

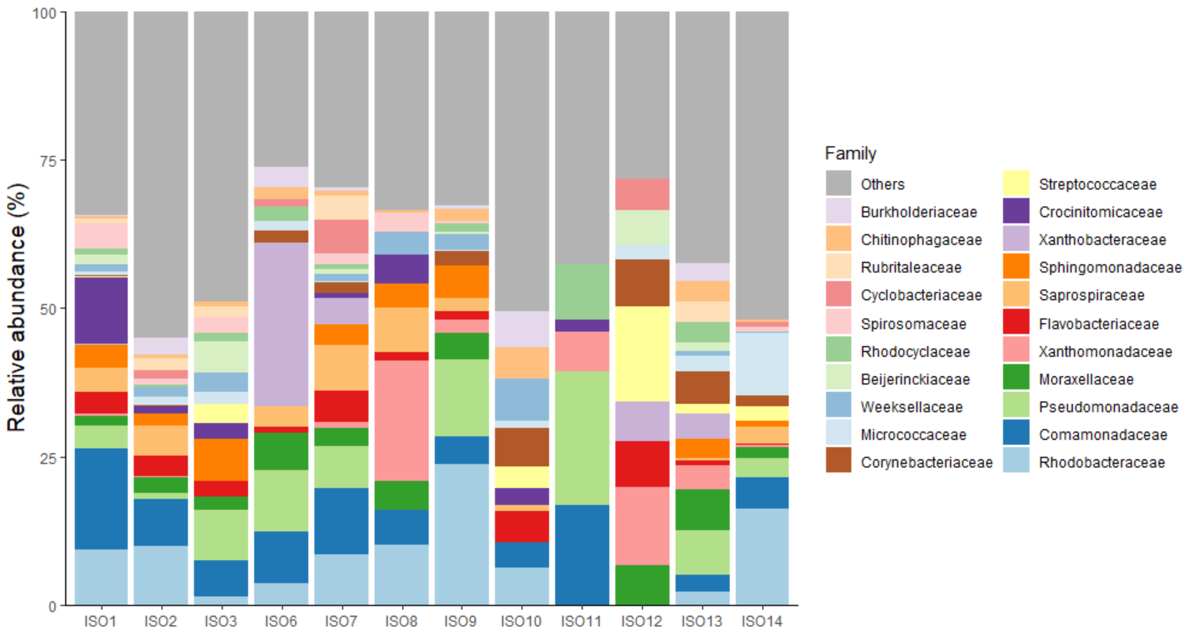

(b)

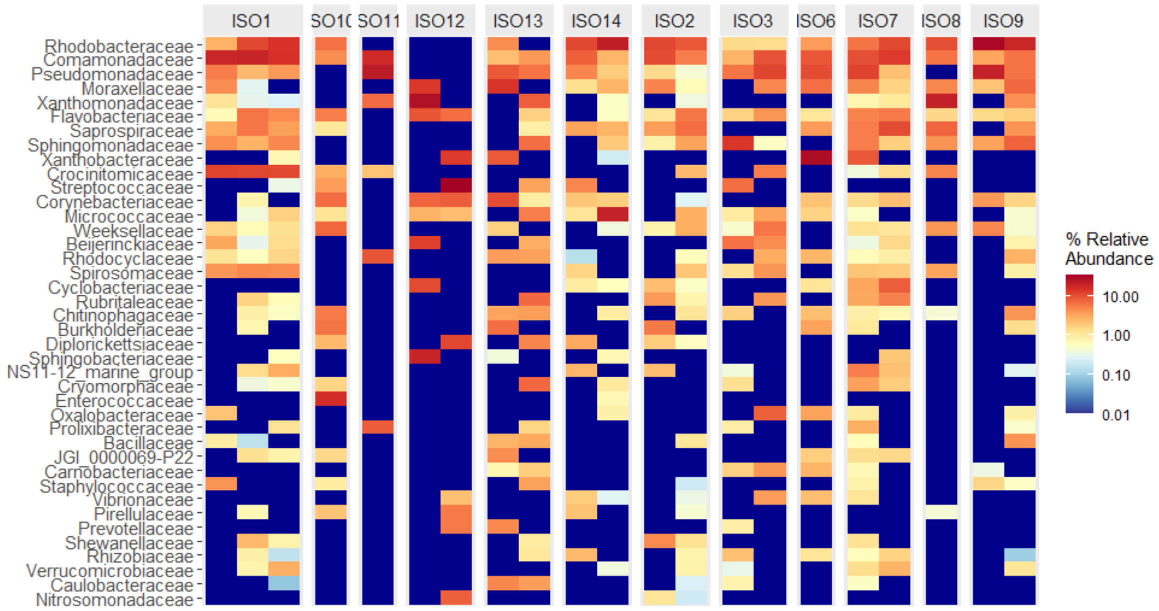

Figure S4. Relative abundance of microbiome (bacterial taxa) of *Maritrema poulini* sampled from isopod hosts at family level. (a) Relative abundance bar plots, x axis is each isopod host, each bar plot represents mean relative abundance of bacteria at family level of all *M. poulini* individuals from same isopod hosts; (b) heatmaps for relative abundance, each column represents relative abundance of bacteria at family level of each *M. poulini* sample.

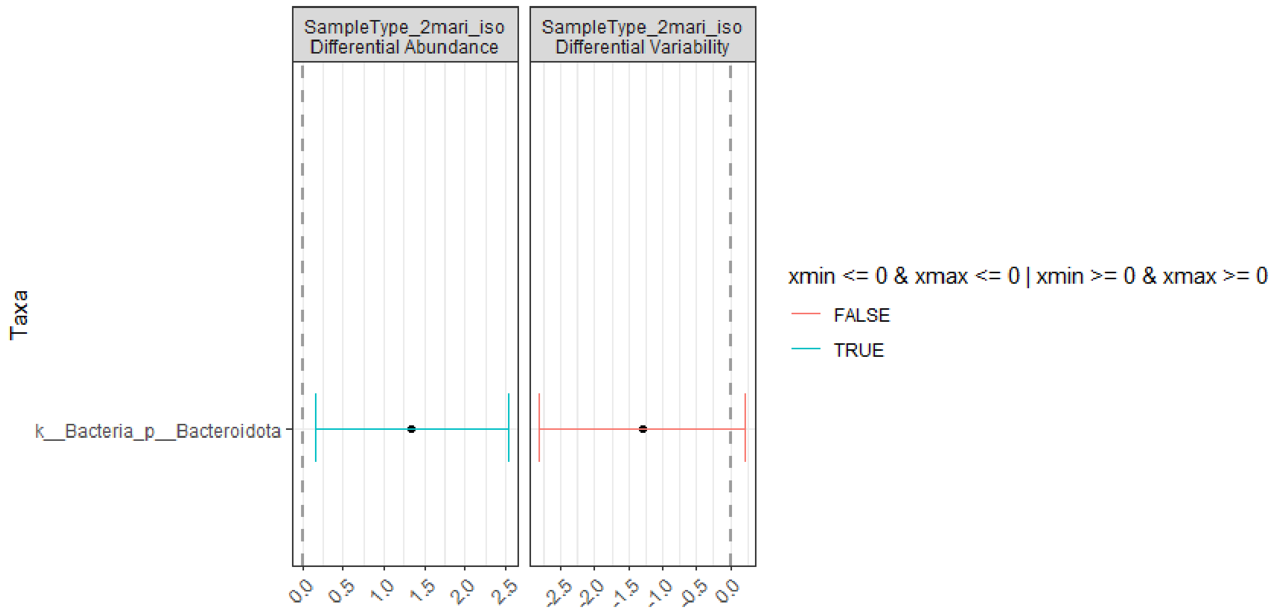

Figure S5. Bacterial phylum in the *Maritrema poulini* microbiome that was in significant differential abundance (higher abundance in *Maritrema* from isopods), based on the Corncob test, between parasites sampled from amphipod and isopod hosts. True (blue interval) indicates statistical significance, and false (red interval) indicates statistical non-significance. X axis of the plot indicates the value of the differential abundance and differential variability for *Maritrema* from isopods (SampleType_2mari_iso).

(a)

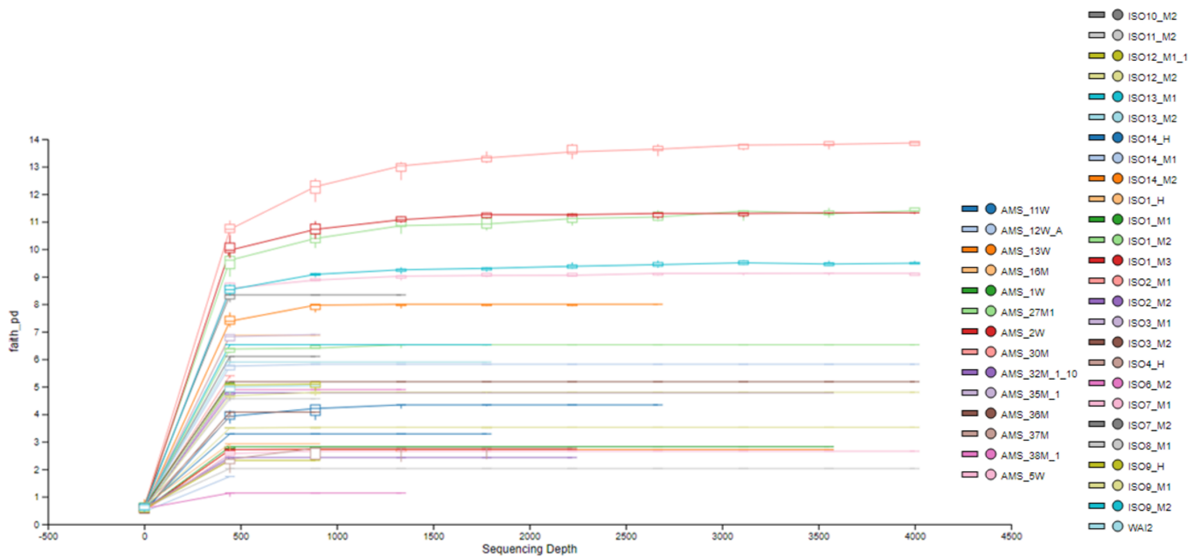
faith_pd
Sequencing Depth
-500
0
500
1000
1500
2000
2500
3000
3500
4000
4500
0
1
2
3
4
5
6
7
8
9
10
11
12
13
14
AMS_11W
AMS_12W_A
AMS_13W
AMS_16M
AMS_1W
AMS_27M1
AMS_2W
AMS_30M
AMS_32M_1_10
AMS_35M_1
AMS_36M
AMS_37M
AMS_38M_1
AMS_5W
ISO10_M2
ISO11_M2
ISO12_M1_1
ISO12_M2
ISO13_M1
ISO13_M2
ISO14_H
ISO14_M1
ISO14_M2
ISO1_H
ISO1_M1
ISO1_M2
ISO1_M3
ISO2_M1
ISO2_M2
ISO3_M1
ISO3_M2
ISO4_H
ISO6_M2
ISO7_M1
ISO7_M2
ISO8_M1
ISO9_H
ISO9_M1
ISO9_M2
WAI2

(b)

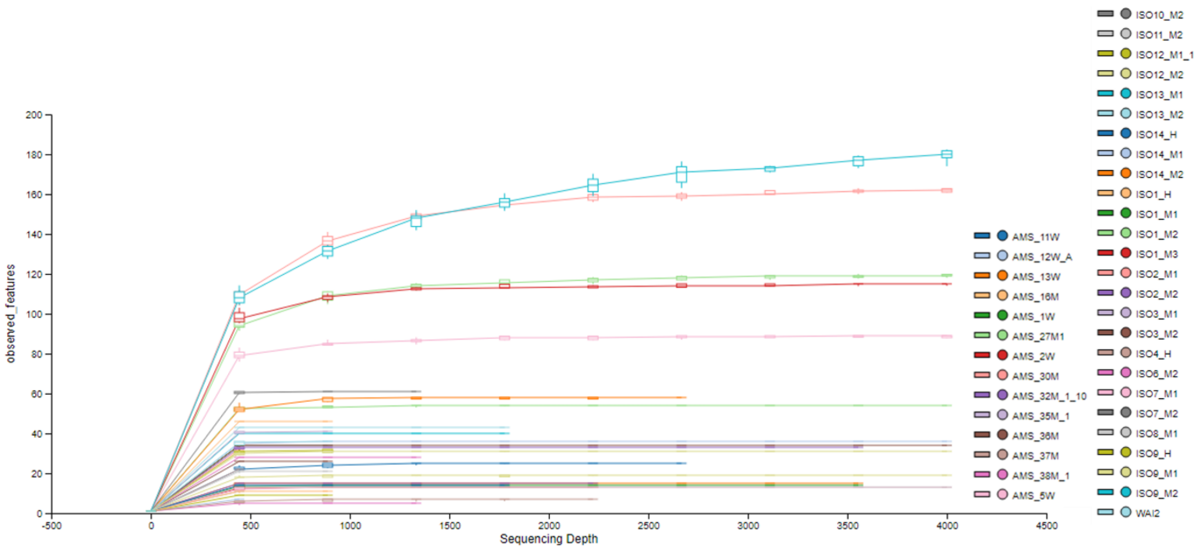
observed_features
Sequencing Depth
-500
0
500
1000
1500
2000
2500
3000
3500
4000
4500
0
20
40
60
80
100
120
140
160
180
200
AMS_11W
AMS_12W_A
AMS_13W
AMS_16M
AMS_1W
AMS_27M1
AMS_2W
AMS_30M
AMS_32M_1_10
AMS_35M_1
AMS_36M
AMS_37M
AMS_38M_1
AMS_5W
ISO10_M2
ISO11_M2
ISO12_M1_1
ISO12_M2
ISO13_M1
ISO13_M2
ISO14_H
ISO14_M1
ISO14_M2
ISO1_H
ISO1_M1
ISO1_M2
ISO1_M3
ISO2_M1
ISO2_M2
ISO3_M1
ISO3_M2
ISO4_H
ISO6_M2
ISO7_M1
ISO7_M2
ISO8_M1
ISO9_H
ISO9_M1
ISO9_M2
WAI2

(c)

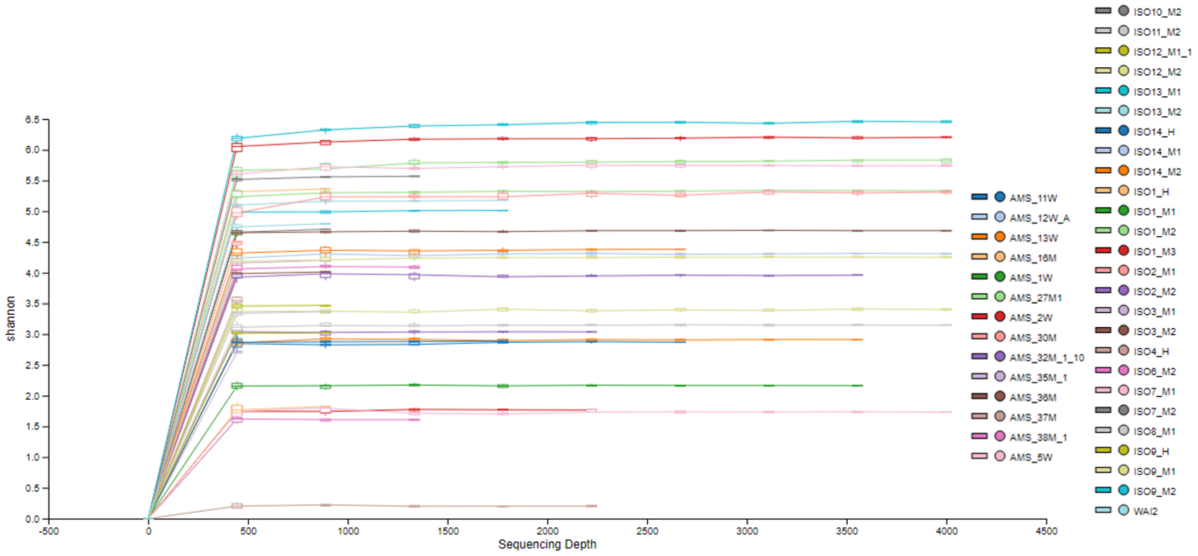

Figure S6. Rarefaction curves posterior to depth filtering. (a) Faith’s PD alpha diversity vs. sequencing depth; (b) Observed features vs. sequencing depth. (c) Shannon diversity vs. sequencing depth. AMS = amphipod; 41 ISO = isopod; W =host whole body tissue; M = *Maritrema poulini*; WAI= Lake Waihola environmental sample (lake water).

Table S1. Sequencing results showing the initial concentration of each library as measured by Qubit in ng/ul and the Yield after sequencing (in mega bases), as well as the mean Phred quality score for each sample.

Library ID	Sample ID	Qubit (ng/ul)	Primers	Golay Barcode	Lane	Project	Sample	Barcode sequence	Yield (Mbases)	Mean Quality Score
OG8441_001	AMS_12W_A	14.2	515rcbc0	AGCCTTCGTCGC	1	OG8441	8441-01-00-01	AGCCTTCGTCGC	1	36.1
OG8441_002	AMS_14W	4.72	515rcbc12	CGTATAAATGCG	1	OG8441	8441-02-00-01	CGTATAAATGCG	0	35.05
OG8441_003	ISO1_H	45.9	515rcbc24	TGACTAATGGCC	1	OG8441	8441-03-00-01	TGACTAATGGCC	1	35.96
OG8441_004	ISO1_M1	0.974	515rcbc36	GTGGAGTCTCAT	1	OG8441	8441-04-00-01	GTGGAGTCTCAT	1	36.14
OG8441_005	ISO1_M2	0.059	515rcbc48	TGATGTGCTAAG	1	OG8441	8441-05-00-01	TGATGTGCTAAG	12	29.45
OG8441_006	ISO1_M3	TOO LOW	515rcbc60	TGTGCACGCCAT	1	OG8441	8441-06-00-01	TGTGCACGCCAT	16	29.17
OG8441_007	POSITIVE	1.82	515rcbc72	GGTGAGCAAGCA	1	OG8441	8441-07-00-01	GGTGAGCAAGCA	3	36.15
OG8441_008	NEGATIVE_190423	TOO LOW	515rcbc84	CTATGTATTAGT	1	OG8441	8441-08-00-01	CTATGTATTAGT	2	21
OG8441_009	AMS_1W	34.1	515rcbc1	TCCATACCGGAA	1	OG8441	8441-09-00-01	TCCATACCGGAA	3	35.66
OG8441_010	AMS_13W	29.4	515rcbc13	ATGCTGCAACAC	1	OG8441	8441-10-00-01	ATGCTGCAACAC	2	36.18
OG8441_011	AMS_13M2	1.59	515rcbc25	CGGGACACCCGA	1	OG8441	8441-11-00-01	CGGGACACCCGA	1	35.73
OG8441_012	AMS_16W	12.4	515rcbc37	ACCTTACACCTT	1	OG8441	8441-12-00-01	ACCTTACACCTT	0	32.35
OG8441_013	AMS_16M	1.21	515rcbc49	GTAGTAGACCAT	1	OG8441	8441-13-00-01	GTAGTAGACCAT	1	35.74
OG8441_014	AMS_22W	10.8	515rcbc61	CCGGACAAGAAG	1	OG8441	8441-14-00-01	CCGGACAAGAAG	0	36.32
OG8441_015	AMS_22M	2.13	515rcbc73	TAAATATACCCT	1	OG8441	8441-15-00-01	TAAATATACCCT	1	35.54
OG8441_016	NEGATIVE_160623	0.154	515rcbc85	ACTCCCGTGTGA	1	OG8441	8441-16-00-01	ACTCCCGTGTGA	18	33.52
OG8441_017	AMS_2W	34.6	515rcbc96	CTACAGGGTCTC	1	OG8441	8441-17-00-01	CTACAGGGTCTC	1	36.18
OG8441_018	NEGATIVE_PULL	0.547	515rcbc180	TCATTAGCGTGG	1	OG8441	8441-18-00-01	TCATTAGCGTGG	2	32.06
OG8441_019	AMS_4W	3.62	515rcbc109	GTGCCGGCCGAC	1	OG8441	8441-19-00-01	GTGCCGGCCGAC	0	35.79

OG8441_020	AMS_5W	36.6	515rcbc121	AGAGGGTGATCG	1	OG8441	8441-20-00-01	AGAGGGTGATCG	3	36.11
OG8441_021	AMS_6W	7.17	515rcbc133	GGATACTCGCAT	1	OG8441	8441-21-00-01	GGATACTCGCAT	0	35.69
OG8441_022	AMS_7W	14.8	515rcbc145	TCATGGCCTCCG	1	OG8441	8441-22-00-01	TCATGGCCTCCG	0	35.93
OG8441_023	AMS_8W	6.63	515rcbc157	ATGTGCTGCTCG	1	OG8441	8441-23-00-01	ATGTGCTGCTCG	0	36.52
OG8441_024	ISO2_M2	0.388	515rcbc169	CCACGAGCAGGC	1	OG8441	8441-24-00-01	CCACGAGCAGGC	2	36.01
OG8441_025	ISO3_H	48.9	515rcbc181	CGCCGTACTTGC	1	OG8441	8441-25-00-01	CGCCGTACTTGC	0	35.49
OG8441_026	AMS_9W	24.1	515rcbc98	TATCATATTACG	1	OG8441	8441-26-00-01	TATCATATTACG	0	36.71
OG8441_027	AMS_10W	8.11	515rcbc110	CCTTGACCGATG	1	OG8441	8441-27-00-01	CCTTGACCGATG	0	34.98
OG8441_028	AMS_11W	7.71	515rcbc122	AGCTCTAGAAAC	1	OG8441	8441-28-00-01	AGCTCTAGAAAC	2	35.88
OG8441_029	AMS_12W_B	0.787	515rcbc134	AATGTTCAACTT	1	OG8441	8441-29-00-01	AATGTTCAACTT	0	35.54
OG8441_030	ISO2_H	55	515rcbc146	CAATCATAGGTG	1	OG8441	8441-30-00-01	CAATCATAGGTG	0	35.76
OG8441_031	ISO2_M1	0.112	515rcbc158	CCGATAAAGGTT	1	OG8441	8441-31-00-01	CCGATAAAGGTT	21	28.42
OG8441_032	ISO3_M1	1.54	515rcbc170	GCGTGCCCGGCC	1	OG8441	8441-32-00-01	GCGTGCCCGGCC	1	36.02
OG8441_033	ISO3_M2	0.423	515rcbc182	TAAACCTGGACA	1	OG8441	8441-33-00-01	TAAACCTGGACA	4	35.73
OG8441_034	AMS_15W	2.45	515rcbc99	CTATATTATCCG	1	OG8441	8441-34-00-01	CTATATTATCCG	0	35.4
OG8441_035	AMS_27W	34	515rcbc111	CAAACTGCGTTG	1	OG8441	8441-35-00-01	CAAACTGCGTTG	0	37.41
OG8441_036	AMS_27M1	0.18	515rcbc123	CTGACACGAATA	1	OG8441	8441-36-00-01	CTGACACGAATA	15	26.29
OG8441_037	AMS_30W	28.8	515rcbc135	AGCAGTGCGGTG	1	OG8441	8441-37-00-01	AGCAGTGCGGTG	0	36.22
OG8441_038	AMS_30M	0.982	515rcbc147	GTTGGACGAAGG	1	OG8441	8441-38-00-01	GTTGGACGAAGG	1	35.29
OG8441_039	AMS_31W	8.58	515rcbc159	CAGGAACCAGGA	1	OG8441	8441-39-00-01	CAGGAACCAGGA	0	35.85
OG8441_040	AMS_31M_1_10	4.19	515rcbc171	CAAAGGAGCCCG	1	OG8441	8441-40-00-01	CAAAGGAGCCCG	2	23.75
OG8441_041	AMS_32W	3.2	515rcbc183	CCAACCCAGATC	1	OG8441	8441-41-00-01	CCAACCCAGATC	0	34.92

OG8441_042	AMS_32M_1_10	3.86	515rcbc100	ACCGAACAATCC	1	OG8441	8441-42-00-01	ACCGAACAATCC	24	21.87
OG8441_043	AMS_33W	32.7	515rcbc112	TCGAGAGTTTGC	1	OG8441	8441-43-00-01	TCGAGAGTTTGC	0	30.55
OG8441_044	AMS_33M1_1	6.22	515rcbc124	GCTGCCCACCTA	1	OG8441	8441-44-00-01	GCTGCCCACCTA	0	25.18
OG8441_045	AMS_35W	38.8	515rcbc136	GCATATGCACTG	1	OG8441	8441-45-00-01	GCATATGCACTG	0	34.92
OG8441_046	AMS_35M_1	5.9	515rcbc148	GTCACTCCGAAC	1	OG8441	8441-46-00-01	GTCACTCCGAAC	2	26.13
OG8441_047	AMS_36W	4.65	515rcbc160	GCATAAACGACT	1	OG8441	8441-47-00-01	GCATAAACGACT	0	
OG8441_048	AMS_36M	0.698	515rcbc172	TGCGGCGTCAGG	1	OG8441	8441-48-00-01	TGCGGCGTCAGG	1	35.29
OG8441_049	AMS_37W	8.99	515rcbc184	TTAAGTTAAGTT	1	OG8441	8441-49-00-01	TTAAGTTAAGTT	0	33.5
OG8441_050	AMS_37M	1.47	515rcbc101	ACGGTACCCTAC	1	OG8441	8441-50-00-01	ACGGTACCCTAC	1	36.07
OG8441_051	AMS_38W	5.67	515rcbc113	CGACACGGAGAA	1	OG8441	8441-51-00-01	CGACACGGAGAA	0	36.29
OG8441_052	AMS_38M_1	3.62	515rcbc125	GCGTTTGCTAGC	1	OG8441	8441-52-00-01	GCGTTTGCTAGC	18	21.73
OG8441_053	AMS_40W	7.3	515rcbc137	CCGGCGACAGAA	1	OG8441	8441-53-00-01	CCGGCGACAGAA	0	34.95
OG8441_054	AMS_40M	0.488	515rcbc149	CGTTCTGGTGGT	1	OG8441	8441-54-00-01	CGTTCTGGTGGT	0	35.58
OG8441_055	AMS_41W	32.2	515rcbc161	ATCGTAGTGGTC	1	OG8441	8441-55-00-01	ATCGTAGTGGTC	0	35.88
OG8441_056	AMS_41M	3.75	515rcbc173	CGCTGTGGATTA	1	OG8441	8441-56-00-01	CGCTGTGGATTA	0	35.92
OG8441_057	ISO4_H	29.1	515rcbc185	AGCCGCGGGTCC	1	OG8441	8441-57-00-01	AGCCGCGGGTCC	2	36.09
OG8441_058	ISO4_M1	2.23	515rcbc102	TGAGTCATTGAG	1	OG8441	8441-58-00-01	TGAGTCATTGAG	0	35.87
OG8441_059	ISO4_M2	2.04	515rcbc114	TCCACAGGGTTC	1	OG8441	8441-59-00-01	TCCACAGGGTTC	0	36
OG8441_060	ISO5_H	47.9	515rcbc126	AGATCGTGCCTA	1	OG8441	8441-60-00-01	AGATCGTGCCTA	0	36.14
OG8441_061	ISO5_M1	2.14	515rcbc138	CCTCACTAGCGA	1	OG8441	8441-61-00-01	CCTCACTAGCGA	0	35.52
OG8441_062	ISO5_M2	5.72	515rcbc150	TAGTTCGGTGAC	1	OG8441	8441-62-00-01	TAGTTCGGTGAC	0	34.16
OG8441_063	ISO6_H	36.4	515rcbc162	ACTAAAGCAAAC	1	OG8441	8441-63-00-01	ACTAAAGCAAAC	0	35.02

OG8441_064	ISO6_M1	2.1	515rcbc174	CTTGCTCATAAT	1	OG8441	8441-64-00-01	CTTGCTCATAAT	0	35.42
OG8441_065	ISO6_M2	2.5	515rcbc186	GGTAGTTCATAG	1	OG8441	8441-65-00-01	GGTAGTTCATAG	5	35.26
OG8441_066	ISO7_H	45.4	515rcbc103	ACCTACTTGTCT	1	OG8441	8441-66-00-01	ACCTACTTGTCT	0	35.83
OG8441_067	ISO7_M1	1.03	515rcbc115	GGAGAACGACAC	1	OG8441	8441-67-00-01	GGAGAACGACAC	25	35.96
OG8441_068	ISO7_M2	0.707	515rcbc127	AATTAATATGTA	1	OG8441	8441-68-00-01	AATTAATATGTA	1	35.82
OG8441_069	ISO8_H	52	515rcbc139	CTAATCAGAGTG	1	OG8441	8441-69-00-01	CTAATCAGAGTG	0	36.15
OG8441_070	ISO8_M1	3.89	515rcbc151	TTAATGGATCGG	1	OG8441	8441-70-00-01	TTAATGGATCGG	1	34.69
OG8441_071	ISO8_M2	1.45	515rcbc163	TAGGAACTCACC	1	OG8441	8441-71-00-01	TAGGAACTCACC	26	21.09
OG8441_072	ISO9_H	37.2	515rcbc175	ACGACAACGGGC	1	OG8441	8441-72-00-01	ACGACAACGGGC	1	35.31
OG8441_073	ISO9_M1	0.481	515rcbc187	CGATGAATATCG	1	OG8441	8441-73-00-01	CGATGAATATCG	7	33.29
OG8441_074	ISO9_M2	TOO LOW	515rcbc104	ACTGTGACGTCC	1	OG8441	8441-74-00-01	ACTGTGACGTCC	513	32.55
OG8441_075	ISO10_H	30.4	515rcbc116	CCTACCATTGTT	1	OG8441	8441-75-00-01	CCTACCATTGTT	0	35.32
OG8441_076	ISO10_M1_1_100	5.06	515rcbc128	CATTTCGCACTT	1	OG8441	8441-76-00-01	CATTTCGCACTT	0	24.66
OG8441_077	ISO10_M2	1.05	515rcbc140	CTACTCCACGAG	1	OG8441	8441-77-00-01	CTACTCCACGAG	1	35.25
OG8441_078	ISO11_H	51	515rcbc152	TCAAGTCCGCAC	1	OG8441	8441-78-00-01	TCAAGTCCGCAC	0	35.36
OG8441_079	ISO11_M1_1_100	4.58	515rcbc164	GTCCGTCCTGGT	1	OG8441	8441-79-00-01	GTCCGTCCTGGT	0	23.55
OG8441_080	ISO11_M2	0.189	515rcbc176	CTAGCGTGCGTT	1	OG8441	8441-80-00-01	CTAGCGTGCGTT	32	24.42
OG8441_081	ISO12_H	46.2	515rcbc188	GTTCTAAGGTGA	1	OG8441	8441-81-00-01	GTTCTAAGGTGA	0	35.91
OG8441_082	ISO12_M1_1	3.64	515rcbc105	CTCTGAGGTAAC	1	OG8441	8441-82-00-01	CTCTGAGGTAAC	48	21.38
OG8441_083	ISO12_M2	0.176	515rcbc117	TCCGGCGGGCAA	1	OG8441	8441-83-00-01	TCCGGCGGGCAA	36	25.74
OG8441_084	ISO13_H	28.9	515rcbc129	ACATGATATTCT	1	OG8441	8441-84-00-01	ACATGATATTCT	0	35.85
OG8441_085	ISO13_M1	0.326	515rcbc141	TAAGGCATCGCT	1	OG8441	8441-85-00-01	TAAGGCATCGCT	2	33.46

OG8441_086	ISO13_M2	0.48	515rcbc153	CACACAAAGTCA	1	OG8441	8441-86-00-01	CACACAAAGTCA	2	33.88
OG8441_087	ISO14_H	0.625	515rcbc165	CGAGGCGAGTCA	1	OG8441	8441-87-00-01	CGAGGCGAGTCA	3	29.96
OG8441_088	ISO14_M1	0.389	515rcbc177	TAGTCTAAGGGT	1	OG8441	8441-88-00-01	TAGTCTAAGGGT	4	34.27
OG8441_089	ISO14_M2	1.62	515rcbc189	ATGACTAAGATG	1	OG8441	8441-89-00-01	ATGACTAAGATG	2	35.84
OG8441_090	WAI1_1	9.23	515rcbc106	CATGTCTTCCAT	1	OG8441	8441-90-00-01	CATGTCTTCCAT	7	21.34
OG8441_091	WAI2	15.9	515rcbc118	TAATCCATAATC	1	OG8441	8441-91-00-01	TAATCCATAATC	1	36.14
OG8441_092	LW1	25.6	515rcbc130	GCAACGAACGAG	1	OG8441	8441-92-00-01	GCAACGAACGAG	0	35.46
OG8441_093	LW2	41.6	515rcbc142	AGCGCGGCGAAT	1	OG8441	8441-93-00-01	AGCGCGGCGAAT	0	36.48
OG8441_094	PBS1	0.389	515rcbc154	GTCAGGTGCGGC	1	OG8441	8441-94-00-01	GTCAGGTGCGGC	15	27.29
OG8441_095	DNAneg_pooled	0.359	515rcbc166	TTCCAATACTCA	1	OG8441	8441-95-00-01	TTCCAATACTCA	4	33.75
OG8441_P1	Pooled_lib	0.6	na	na	1	default	Undetermined	unknown	922	35.33

122

Table S2 PerMANOVAs and betadisper results for the comparison of the microbiota of *Maritrema poulini* sampled within the same and among different isopod hosts. Significant perMANOVAs results that did not violate the multivariate homogeneity of groups' dispersions are shown in bold.

Taxonomic level	Beta diversity metrics	PerMANOVA result	betadisper
phylum	Bray-Curtis	non-significant	NA
	Jaccard	P=0.034	P=0.001
	Weighted Unifrac	non-significant	NA
	Unweighted Unifrac	P=0.035	P=0.001
family	Bray-Curtis	P=0.001	P=0.001
	Jaccard	**P=0.004**	Non-significant
	Weighted Unifrac	P=0.002	P=0.001
	Unweighted Unifrac	P=0.004	P=0.001
ASV	Bray-Curtis	P=0.001	P=0.001
	Jaccard	**P=0.001**	Non-significant
	Weighted Unifrac	P=0.002	P=0.001
	Unweighted Unifrac	**P=0.004**	Non-significant

Table S3. PerMANOVAs and betadisper results of microbiome of *Maritrema poulini* sampled between amphipod and isopod hosts.

Taxonomic level	Beta diversity metrics	PerMANOVA result	betadisper
phylum	Bray-Curtis	p.adjust=0.011	Non significant
	Jaccard	Non significant	NA
	Weighted Unifrac	P=0.002	Non significant
	Unweighted Unifrac	Non significant	NA
family	Bray-Curtis	p.adjust=0.001	Non significant
	Jaccard	p.adjust=0.001	Non significant
	Weighted Unifrac	p.adjust=0.001	Non significant
	Unweighted Unifrac	p.adjust=0.005	Non significant
ASV	Bray-Curtis	p.adjust=0.002	Non significant
	Jaccard	p.adjust=0.001	Non significant
	Weighted Unifrac	p.adjust=0.001	Non significant
	Unweighted Unifrac	p.adjust=0.003	Non significant

Table S4. Sample ID for amphipods and Maritrema poulini sampled from amphipods used in Venn diagram and PerMANOVA test.

Amphipods sample ID	*Maritrema poulini* sample ID
AMS_12 (uninfected)	AMS_16M
AMS_1 (uninfected)	AMS_27M
AMS_13 (infected)	AMS_30M
AMS_2 (uninfected)	AMS_32M
AMS_5 (uninfected)	AMS_35M
AMS_11 (uninfected)	AMS_36M
	AMS_37M
	AMS_38M

Table S5. PerMANOVA and betadisper results of microbiome of amphipods and *Maritrema poulini* sampled from amphipod hosts.

Taxonomic level	Beta diversity metrics	PerMANOVA result	betadisper
ASV	Bray-Curtis	P = 0.002	P = 0.001
	Jaccard	P = 0.001	P = 0.001
	Weighted Unifrac	P = 0.001	Non significant
	Unweighted Unifrac	P = 0.001	P = 0.008

Table S6. Sample ID for amphipods and *Maritrema poulini* sampled from amphipods used in Venn diagram and PerMANOVA test.

Isopods sample ID	*Maritrema poulini* sample ID
ISO1_H	ISO1_M1
ISO4_H	ISO1_M2
ISO9_H	ISO1_M3
ISO14_H	ISO2_M2
	ISO2_M1
	ISO3_M1
	ISO3_M2
	ISO6_M2
	ISO7_M1
	ISO7_M2
	ISO8_M1
	ISO9_M1
	ISO9_M2
	ISO10_M2
	ISO11_M2
	ISO12_M1
	ISO12_M2
	ISO13_M1
	ISO13_M2
	ISO14_M1
	ISO14_M2

Table S7. PerMANOVA and betadisper results of microbiome of isopods and *Maritrema poulini* sampled from isopod hosts.

Taxonomic level	Beta diversity metrics	PerMANOVA result	betadisper
ASV	Bray-Curtis	P = 0.208	P = 0.001
	Jaccard	P = 0.122	P = 0.001
	Weighted Unifrac	P = 0.002	Non significant
	Unweighted Unifrac	P = 0.025	P = 0.002
